# Protocol for exploring health promoter-led mental wellness initiatives for early prevention, screening and quality of life in patients with cervical cancer of rural Eastern Cape, South Africa: a mixed-methods study

**DOI:** 10.1136/bmjopen-2025-104827

**Published:** 2026-03-25

**Authors:** Khuthala Sigovana, Sibusiso C Nomatshila, Wezile Chitha, Sikhumbuzo A Mabunda

**Affiliations:** 1 School of Public Health, Walter Sisulu University Faculty of Health Sciences, Mthatha, South Africa; 2 Walter Sisulu University, Mthatha, South Africa; 3 School of Public Health, Society and Health Research Institute, Walter Sisulu University, Mthatha, South Africa; 4 Institution for Clinical Governance and Health Care Administration, Walter Sisulu University Faculty of Health Sciences, Mthatha, South Africa; 5 George Institute for Global Health, University of New South Wales, Sydney, New South Wales, Australia

**Keywords:** MENTAL HEALTH, Early Detection of Cancer, Cancer pain, Community-Based Participatory Research, Health Education, Psychosocial Intervention

## Abstract

**Introduction:**

Cervical cancer is in the top five list of highly prevalent cancers. It contributes to millions of premature deaths globally. Low- and middle-income countries like those in sub-Saharan Africa are among the worst affected despite the preventable nature of the disease. The disease is caused by the human papillomavirus, a sexually transmitted infection for which an effective vaccine exists. This study aims to determine the effectiveness of a health promoter-led intervention on early screening, prevention and quality-of-life improvement among patients with cervical cancer.

**Methods and analysis:**

The study will be conducted in South Africa’s Eastern Cape province. The study will employ an exploratory sequential mixed-methods design, comprising four qualitative and quantitative substudies. For qualitative substudies, purposive sampling will select 20 cervical patients, and two to four focus group discussions will be conducted with health workers in the oncology units of two referral hospitals. Patients with a cervical cancer diagnosis will consent to participate in a survey on their quality of life and their mental health status, specifically screening for depression and anxiety. An equal number of patients (64) will be allocated to the two quantitative study arms. For substudies 1 and 2, in-depth interviews and focus group discussions will be conducted using a semistructured interview guide. The data will be audio recorded, transcribed verbatim and entered into NVivo V.15 for data analysis. For substudies 3 and 4, data will be collected using validated tools such as the WHO-Quality of Life tool to assess the quality of life of cervical cancer survivors, the Generalized Anxiety Disorder version 7 scale and the Patient Health Questionnaire version 9 for anxiety and depression. Data will be captured in Microsoft Excel and exported to SPSS V.29 software (Statistical Package for the Social Sciences). The study will use descriptive analyses of intervention participants’ responses using means, SDs, medians and IQRs, depending on the normality of the distribution.

**Ethics and dissemination:**

The ethical clearance (protocol reference number: WSU HREC 023/2025) was approved by the Walter Sisulu University, Faculty of Medicine and Health Sciences, Ethics and Biosafety Committee, and access approval (reference number: EC_202503_022) was granted by the Eastern Cape Provincial Health Research Committee.

**Trial registration number:**

PACTR202507608317068.

**STRENGTHS AND LIMITATIONS OF THIS STUDY**

- To our knowledge, this is the first health promoter-led intervention study on cervical cancer and quality of life in the low- and middle-income country context.
- The study will focus on underserved rural communities and the holistic approach to health that integrates mental wellness with cervical cancer prevention.
- The study has the potential to empower communities through the use of health promoters on mental health and cervical cancer, as it uses principles of community engagement and involvement throughout its implementation.
- The paucity of literature on the subject and its context means there will be a lot of trial and error in the intervention to be explored.
- Since the study will only be conducted for 4 months and the intervention will be short term, it will be difficult to establish the sustainability of the improvements in mental health and quality of life among participants.

## Background

 According to the 2022 global statistics, cervical cancer (CC) is the fourth most common cancer in terms of both incidence and mortality in women, with an estimated 660 000 new cases and 350 000 deaths worldwide.^1^ Statistics from the WHO show that the incidence and mortality rates of CC are increasing in the African continent, with 125 699 new CC cases recorded, including Northern Africa, Middle Africa, Western Africa, Southern Africa and Eastern Africa.^2^ With over 80% of the worldwide CC burden occurring in low- and middle-income countries (LMICs), the disease disproportionately affects these nations, with sub-Saharan Africa (SSA) having the highest age-standardised incidence and mortality rates in 2018.^3^ The top five regions with the most significant incidence and mortality rates of CC cases are Eastern, Southern, Middle, Western and Northern Africa.^4 5^ According to Amponsah-Dacosta *et al*,^6^ the incidence and mortality rates of CC in South Africa are among the highest in SSA. Out of the 117 316 new cases and 76 745 deaths documented in the African region, 10 702 (9.1%) CC cases and 5870 (7.6%) associated fatalities were reported from South Africa alone in 2020. Statistics from the WHO show that the incidence and mortality rates of CC are increasing in the African continent, with 125 699 new CC cases recorded, including Northern Africa, Middle Africa, Western Africa, Southern Africa and Eastern Africa.^2 3^

CC has also been confirmed as the leading cause of cancer-related deaths among women in Africa and Central America.^5^ CC is a critical public health issue in SSA, where it is the second leading cause of cancer among women and a leading cause of female deaths.^5 7^ According to Global Cancer Statistics, SSA is the highest region with elevated rates of incidence and mortality in Eastern, Southern and Central Africa.^4^

High-risk subtypes 16 and 18 of the human papillomavirus (HPV) are the cause of the disease in at least 70% of CC cases.^7^,^12^ These high-risk HPV subtypes 16 and 18 were also detected among South African female high school learners, where the prevalence was 8.5% and 8.0%, respectively.^10^ Other significant contributing factors to CC include smoking, a higher number of pregnancies, long-term oral contraceptive use and some sexually transmitted diseases such as HIV and *Chlamydia trachomatis*.^4^

Higher levels of HPV or numerous HPV types, as well as abnormal cervical cytology, are associated with HIV.^9 12^ Women who have HIV have a higher chance of HPV infection and have a chance of developing an early-onset CC while living with HIV.^9 12^ While the average age of onset is 15–49 years in the general population, for women living with HIV, this usually occurs between 13 and 18 years.^13^

The incidence of 12 983 cases of CC diagnosed in 2018 has been identified as a leading cause of cancer mortality in South Africa, with an estimated 5595 deaths, mostly in women between the ages of 18 and 44, some of whom are HIV positive.^12 14^ According to the Global Cancer Observatory 2022 report, there were 10 532 new cases of CC, accounting for ±17.9% of this illness diagnosed among South African women, which is decreasing slowly when compared with 2018 statistics. Even though the number of new cases is declining, South African CC deaths are highest among women of all ages, with an estimated annual incidence of 10 532 cases and 5976 deaths a year.^2^

As per the 2013–2017 report by the South African Medical Council, St Elizabeth Hospital (SEH) and Nelson Mandela Academic Hospital (NMAH) in the Oliver Reginald (OR) Tambo region, Eastern Cape (EC), had an age-standardised incidence rate of 33.1 per 100 000 people, making it one of the hospitals with the highest number of CC cases recorded.^15^

CC does not initially cause pain, and only occasionally is spotting reported. Because of this lack of symptoms, CC is usually not detected at an early stage unless the person is screened.^16 17^

According to the South African National Department of Health (NDoH), the most effective strategy is to vaccinate against the two most prevalent oncogenic HPV strains, types 16 and 18, to essentially prevent CC.^18^ The existing policy by the South African NDoH recommends that preadolescent girls should receive the HPV vaccine, which offers the best chance of successfully halting the CC epidemic in LMICs.^18^

The HPV vaccination programme was started in South Africa in 2014 as part of the Integrated School Health Programme and is carried out in collaboration with the Departments of Basic Education and Social Development.^18 19^ It has been confirmed that these vaccines work best if administered ahead of exposure to HPV.^12 18 19^ Both vaccines (bivalent HPV vaccine, also known as Cervarix; quadrivalent, known as Gardasil) protect against more than 95% of HPV infections brought on by HPV types 16 and 18, as well as some other less frequent HPV forms that can lead to CC. Furthermore, one of the vaccines (quadrivalent) also offers defence against HPV strains 6 and 11, which are responsible for genital warts.^12 18 19^

In 2020, 75% of 15-year-old South African adolescent girls had received at least one dose of the HPV vaccine at any point between the ages of 9 and 14 years, and 61% had finished the entire recommended two-dose schedule.^6^ These statistics have given some insight into the scope of the HPV vaccination campaign in South Africa during the last 6 years.^6^

In South Africa, the screening policy for CC was established in 2000 and recommends three Pap smear screenings to be performed during one’s lifespan, starting at 30 years of age, occurring at 10-year intervals if the woman is HIV negative.^9^ However, for HIV-positive women, screening must be conducted annually or at 3-year intervals, depending on the screening results, for the remainder of the woman’s life, irrespective of the CD4 cell count and antiretroviral treatment.^18 20^ All low-risk women found to have an abnormality during routine screening should then be screened at 3-year intervals until the screen result is negative.^18 20^

Between 2000 and 2019, the South African screening programme prevented 8600 (95% CI 4700 to 12 300) cases of CC.^21^ According to predictions, age-standardised CC incidence will drop from 49.4 per 100 000 women (95% CI 36.6 to 67.2) in 2020 to 12.0 per 100 000 women (95% CI 8.0 to 17.2) in 2120, with current levels of prevention (status quo vaccine, screening and treatment).^21^ Even though the South African screening programme attempts to prevent CC, only 35.5% of women of reproductive age used Pap smear testing, indicating that most women did not take advantage of CC screening.^22^

### CC and mental health

Mental health conditions such as depression and anxiety are known to be the most prevalent effects associated with CC, and the existence of these disorders is likely to influence CC prognosis by affecting the patient’s quality of life or therapeutic success.^16 23^ In addition, Kim *et al*^23^ noted that women might face more mental health problems than men due to their femininity-related factors when they are diagnosed with gynaecological cancer. A coordinated team of multidisciplinary health personnel is required to manage the patient holistically and comprehensively because a cancer diagnosis is stressful, as it affects the patient physically, psychologically and financially.^24^

A 2022 Chinese study found 16% of enrolled patients with cancer (n=59) to have clinical symptoms of mental disorders, with depression, anxiety, psychotic symptoms and stress-related disorders accounting for 13.3%, 10.2%, 2.8% and 1.4%, respectively.^25^ This is because some patients equate a cancer diagnosis with a death sentence.^25^ As such, the diagnosis can cause anxiety, depression and other psychological problems for many patients.^25^ Patients experience high levels of stress, especially after diagnosis and during treatment initiation, which can put an additional burden on patients.

Notwithstanding, CC screening can also trigger anxiety in some patients.^26^ This is more severe among women with a history of sexual violence and those who experienced sexual assault during their childhood.^26^ These women can find pelvic examination to be a triggering event, thus leading them to avoid screening.^26^

CC treatment includes multiple modalities, including surgery, chemotherapy and radiation, and leads to changes in affected women.^16 27^ According to Klügel *et al*^16^ and Chona *et al*,^28^ women of childbearing age may express mild to severe sadness due to infertility caused by the disease’s therapy. Additionally, a hysterectomy is a medical operation where the uterus is removed from the woman’s body as part of the therapy for CC in some women.^29^ Therefore, the sudden and unexpected change can result in infertility and may worsen mental health conditions, and in some cases, can result in post-traumatic stress disorder for young CC survivors.^29^

About half of the countries in Africa, where the disease burden is highest, are reported to be lacking palliative care services.^30 31^ Palliative care is only provided in remote tertiary facilities that are far from most of the population in the majority of those African countries.^30 31^

Palliative care should be a prerequisite component of CC prevention, early diagnosis, treatment and survivorship care, and it should be offered in all healthcare settings, including in hospitals, long-term care institutions, community health centres and patients’ homes.^32^ Attention must be paid to preventing, quickly diagnosing and treating psychological disorders due to the high prevalence of anxiety, depressive mood, sexual dysfunction and other disorders among women with CC, the suffering they cause and the relationship between untreated psychological disorders and decreased treatment adherence.^32^

There are limited data on the use of health promoters to assist with CC screening and their role in the improvement of CC survivors’ quality of life. Therefore, it is worthwhile determining the role of health promoters in CC prevention and quality-of-life improvement among CC survivors, thereby identifying the associated screening barriers and quality-of-life improvement gaps. This proposed study is expected to bring about new knowledge and innovation related to mental health-conducive screening, treatment initiation and management of CC among community members of OR Tambo District in South Africa. The research will further enhance coping mechanisms and health promoter-led quality of life improvement strategies among patients with CC. The study’s findings will be used to make recommendations to both policymakers and academics on the gaps identified and the possible roles of health promoters in the prevention of CC and the care of CC survivors and their families.

### Objectives of the study

1. To explore the perceived burden of mental health illnesses associated with CC, diagnosis or treatment, targeting patients with CC and healthcare workers in the OR Tambo District.
2. To assess barriers, practices and knowledge on CC screening and HPV vaccination among women and health workers in the EC.
3. To assess the quality of life of patients with a CC diagnosis and further determine factors affecting the patient’s quality of life.
4. To analyse strategies for health promoter-led mental wellness initiatives for early screening and quality-of-life improvement among patients with CC in the OR Tambo District.
5. To conduct a pre-post interventional evaluation of mental health promoter-led early CC screening and treatment initiation.

## Methods and analysis

### Study setting

**Figure 1:**
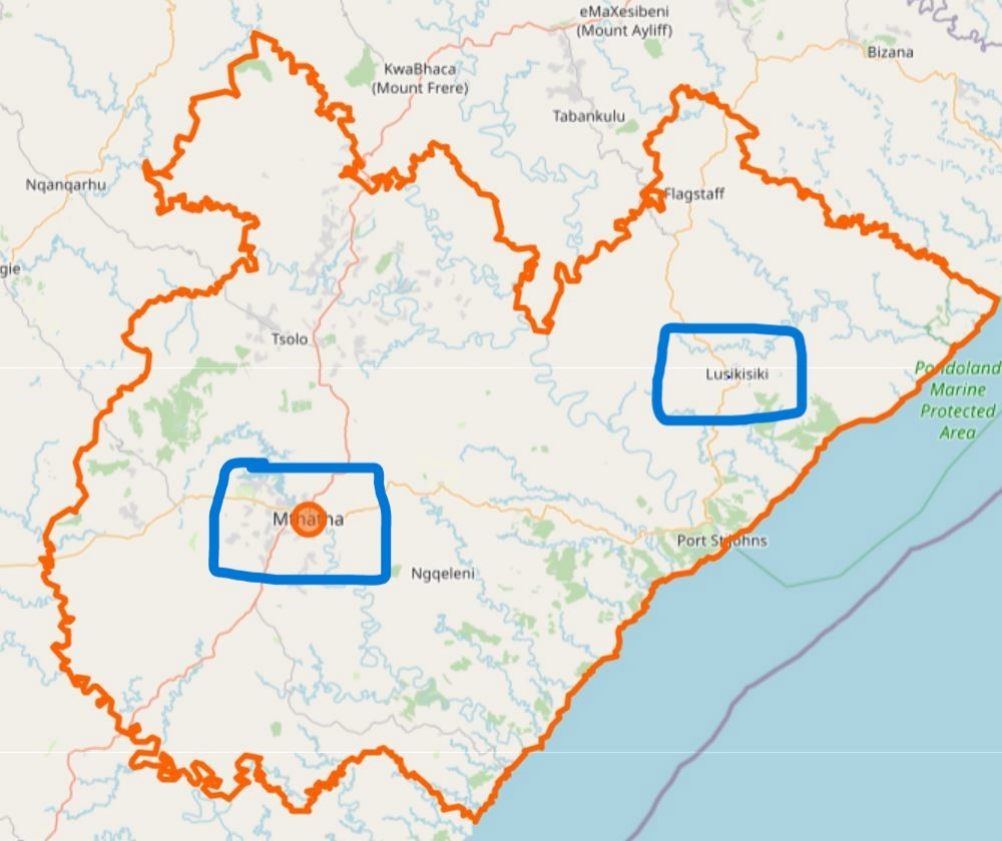
Olive Reginald Tambo District with its subdistricts (*Statistics South Africa, 2023*).

The study will be conducted in OR Tambo District (figure 1) within South Africa’s EC province. The EC is South Africa’s second-biggest province by surface area and the fourth most populous province. The OR Tambo District is the biggest and most populous district, serving five subdistricts in the EC with a population of over 1.5 million.^33^ This research will be undertaken in two hospitals from King Sabata Dalindyebo and Ingquza Hill subdistricts.

### Study design

The study objectives will be achieved through an exploratory sequential mixed-methods design (table 1) with four qualitative and quantitative substudies. The study will have two phases.

**Table 1:** Research methods summary

Study design	Objective	Data points	Data collection method	Data collection instrument	Data analysis
Substudy 1: qualitative study	To assess the quality of life of patients with a cervical cancer diagnosis and further determine factors affecting the patient’s quality of life. To assess barriers, practices and the level of knowledge among Eastern Cape women and health workers on cervical cancer screening and HPV vaccination in the Eastern Cape.	St Elizabeth Hospital and Nelson Mandela Academic Hospital	In-depth interviews	Interview guide	Data will be audio recorded, transcribed verbatim, translated, back translated, corrected for errors and entered into NVivo software for data analysis.
Substudy 2: qualitative study interviews	To investigate the burden of mental health illnesses associated with cervical cancer screening, diagnosis or treatment.	St Elizabeth Hospital and Nelson Mandela Academic Hospital	Focus group discussions	Interview schedule	Data will be audio recorded, transcribed verbatim, translated, back translated, corrected for errors and entered into NVivo V.15 software for data analysis.
Substudies 3 and 4: longitudinal study	To conduct a pre-post interventional evaluation of using a mental health promoter to promote early screening for cervical cancer.	St Elizabeth Hospital and Nelson Mandela Academic Hospital	Questionnaire surveys	Incorporated instrument with elements of WHO-QoL-BREF, GAD-7 and PHQ-9	Data will be captured on Microsoft Excel and exported to SPSS V.29 software.

GAD-7, General Anxiety Disorder version 7; HPV, human papillomavirus; PHQ-9, Patient Health Questionnaire version 9; WHO-QoL-BREF, WHO-Quality of Life-shortened version.

#### Phase I (qualitative substudies)

This phase seeks to establish the baseline situation of CC in two subdistricts.

##### Substudy 1

An in-depth interview substudy that will explore the burden of mental health illnesses associated with CC screening, diagnosis or treatment, and further assess the perceived quality of life among patients with a CC diagnosis.

##### Substudy 2

Focus group discussions (FGD) will be used on healthcare providers to identify individual and system-level barriers to CC screening and vaccination. The study will also ascertain the perceived burden of mental health illnesses associated with CC screening, diagnosis or treatment from health providers.

### Phase II (quantitative longitudinal substudies): randomised controlled trial

#### Substudies 3 and 4 (testing an intervention)

An intervention to improve CC management will be developed based on the findings of phase I substudies. This substudy is reported using the Standard Protocol Items: Recommendations for Interventional Trials checklist (online supplemental appendix 1). This phase will enrol all participants who were identified as having CC. A randomised controlled trial of 128 participants with a CC diagnosis from two hospitals (NMAH and SEH) will be recruited for the intervention. These hospitals will be designated as clusters, with one assigned to the intervention group and the other to the control group through random selection. To ensure allocation concealment and to prevent contamination, the interventions and data collection will be carried out independently in each hospital. Participants will be randomly assigned to two groups, with 64 participants in each of the intervention and control groups. At baseline, quality of life and mental health indicators (anxiety and depression scores: General Anxiety Disorder version 7 (GAD-7) and Patient Health Questionnaire version 9 (PHQ-9)) will be evaluated in both groups as primary outcomes. Secondary outcomes will include attendance rate, retention and data completeness at intervention sessions.

The intervention content will incorporate identified factors from both qualitative (interviews and discussions) and quantitative indicators, such as existing clinical and demographic data, to inform the identification of relevant feasibility factors. The intervention will be implemented by trained health promoters, who will use health literacy, motivational interview techniques and the dissemination of relevant information, education and communication materials regarding CC, mental health and wellness and quality of life. This will be achieved through flip chart boards and the issuance of pamphlets. In the intervention group, four interventions will be implemented at 1-month intervals, each followed by an evaluation of quality of life and mental health outcomes. The four evaluations in the intervention group will run concurrently with those in the control group. The control group will continue to receive the usual care for CC treatment. At the end of the intervention period, the participants with CC in the control group will be revisited for outcome measures. They will further be referred to health facilities for definitive diagnosis and management of their mental health if at risk.

The study will assess differences in mental health indicators at the end of the 4 months (substudy 4).

### Participants and sampling

a. Substudy 1: This will be in-depth interviews with 20 purposively sampled patients, 18 years and above, who have a CC diagnosis at both NMAH and SEH. 10 patients will be consented to at each of the hospitals. The sample size is thought to be adequate for saturation. Saturation is the stage in data collection when no further issues or insights emerge, and the information begins to repeat itself. This indicates that additional data collection is unnecessary and that an adequate sample size has been achieved.^34^,^36^

b. Substudy 2: Depending on the availability of relevant health workers, two to four FGDs will be held at NMAH and SEH with nursing, medical and allied health workers working in the two hospitals’ oncology units. A focus group will have 4–10 members.

c. Substudies 3 and 4: Patients with a CC diagnosis at both NMAH and SEH will be requested to participate in a survey on their quality of life and their mental health status, specifically screening for depression and anxiety. The sample size was calculated using the equation $n=\frac{(\sigma_{1}^{2}+\sigma_{2}^{2})(Z_{1-\alpha }+Z_{1-\beta }{)}^{2}}{(\mu_{1}-\mu_{2}{)}^{2}}$ for a two-sided 95% CI, where α=0.05, Z_1−α_=1.96, and for 80% power, Z_1−β_=0.84. Mean difference µ_1_−µ_2_=1 and SD (σ)=2. The sample size was calculated using a two-sample means formula, assuming a mean difference of 1 and an SD of 2, which corresponds to a medium standardised effect size (Cohen’s d=0.5). This sample size will likely result in a medium effect size due to the nature of the intervention, previous literature and the feasibility and practical impact.

- Nature of the intervention:
  - Health promoter-led initiatives are community based, personalised and culturally sensitive, which tend to yield moderate improvements in mental health and screening uptake.
- Previous literature:
  - Studies on similar interventions (eg, community health worker programmes, psychoeducational support) often report moderate improvements in quality of life, anxiety, depression and screening behaviour.^37^
- Feasibility and practical impact:
  - A medium effect size is clinically meaningful and statistically detectable with a feasible sample size, especially in resource-limited settings like the rural EC. The sample size was calculated to detect a clinically significant difference in the primary clinical outcomes, thereby demonstrating that the study is designed as a fully powered randomised controlled trial rather than a pilot or feasibility study.

Participant recruitment will take place in collaboration with healthcare workers from the oncology units at NMAH and SEH. To raise awareness about the study and its benefits, informational sessions will be conducted within the hospitals, accompanied by posters displayed in key areas, such as the women's health units. Health promoters will actively engage potential participants, providing explanations about the study in local languages and assisting with the informed consent process.

After the initial survey, a cluster randomised controlled trial will be conducted to assess the role of a health promoter-led motivational interview intervention with CC survivors on their mental health status. The primary aim of phases III and IV is to evaluate the effectiveness of the intervention on quality of life and mental health outcomes in a randomised controlled trial, and the second aim is to assess feasibility and implementation-related parameters (eg, recruitment, retention, adherence) rather than to achieve statistical power for hypothesis testing. One of the two hospitals will be randomly assigned to either the control or intervention arm to minimise contamination of the findings (ie, minimise interaction between patient groups). Since the intervention involves health promoter-led mental wellness initiatives, cluster randomising at the hospital level (rather than individual level) helps avoid contamination and aligns with real-world implementation. Step-by-step implementation is outlined below.

1. Define clusters:
  - Each hospital of the two hospitals will serve as a cluster.
  - For this study, clusters will be similar in terms of patient volume.
2. Randomisation procedure:
  - Researchers will use a computer-generated random sequence using STATA statistical software.
  - Randomly assign clusters to either:
    - Intervention group: receives health promoter-led mental wellness initiatives.
    - Control group: receives standard care.
3. Randomisation unit:
  - The unit of randomisation is the hospital, but outcomes are measured at the individual patient level.

*To reduce selection bias*:

- There will be centralised randomisation.
- Researchers will use an independent statistician and data manager to generate and hold the randomisation list.
- Allocation will be revealed only after baseline data collection is complete.
- While full blinding may not be feasible due to the nature of the intervention, outcome assessors and data analysts will be blinded to group allocation.

### Minimising contamination between groups

Contamination occurs when control group participants are exposed to the intervention. This will be achieved through multiple strategies.

1. Physical separation:
  - The hospitals are geographically distant with no crossover of staff.
2. Staff assignment:
  - Assign dedicated health promoters to intervention hospitals only.
  - Avoid rotating staff between intervention and control sites.
3. Communication protocols:
  - Limit sharing of intervention materials or training across clusters until the study concludes.
  - Use separate WhatsApp groups, training sessions and supervision structures for each arm.
4. Monitoring:
  - Conduct periodic checks to ensure fidelity to group assignments.
  - Use surveys to track any unintended exposure to intervention content.

An equal number of patients (64) will be allocated to each of the two study arms. Participants will be excluded if they are attending psychotherapy, have visible psychosis, have suicidal ideation or are not fit to consent. Referral pathways to psychiatric or crisis services are in place for those mental health patients who are excluded, ensuring they are not left without support. Notwithstanding, patients living with CC who have suicidal ideation may benefit from integrated mental health services, and their exclusion could delay access to care. Researchers, therefore, further acknowledge that there are ethical and practical implications of the exclusion criteria on patients with a mental health disorder.

1. Protection versus exclusion:
  - Ethical justification: Excluding individuals with severe mental health conditions is primarily intended to protect them from potential harm, especially if the intervention is not equipped to manage acute psychiatric crises.
  - Practical limitation: However, this exclusion may inadvertently marginalise a subgroup that is both highly vulnerable and potentially in need of the very support the intervention aims to provide.
2. Impact on generalisability:
  - By excluding participants with significant mental health issues, the study may limit the applicability of its findings to the broader population of CC survivors, many of whom experience psychological distress.
  - This could result in underestimating the true burden of mental health challenges in this population and overestimating the effectiveness of the intervention in real-world settings.

The last substudy is the assessment of outcomes between the control and intervention groups to ascertain patients’ mental health status and quality of life using the same instrument as the one used at baseline. To assess whether the intervention is practical, acceptable and scalable, six outcomes will be measured (table 2).

**Table 2:** Prespecified outcomes to measure practicality, acceptability and scalability of the intervention

Domain	Definition	Success criterion	Rationale
Recruitment rate	Proportion of eligible patients with cervical cancer who consent to participate.	≥70% of eligible participants recruited within the planned timeframe.	Indicates community interest and acceptability of health promoter-led initiatives.
Session attendance	Proportion of participants attending ≥75% of scheduled mental wellness sessions.	≥80% of participants meet attendance threshold.	Reflects engagement and feasibility of delivering the intervention in rural settings.
Retention rate	Proportion of participants who complete all study assessments (baseline, postintervention, follow-up).	≥85% retention across study phases.	Ensures data completeness and indicates acceptability of study procedures.
Health promoter fidelity	Adherence to intervention protocols by health promoters.	≥90% of sessions delivered as per protocol (based on checklist or supervision logs).	Confirms intervention integrity and training effectiveness.
Data completeness	Proportion of participants with complete data for primary outcomes (eg, mental health scores, quality of life).	≥90% completeness.	Ensures reliability of outcome measures and feasibility of data collection tools.
Acceptability and satisfaction	Participant-reported satisfaction with the intervention (via postsession surveys or interviews).	≥80% report moderate to high satisfaction.	Gauges cultural and contextual fit of the intervention.

### Data collection tools and procedures

#### Substudy 1

In-person, in-depth interviews will be conducted with CC survivors from the two hospitals using a developed interview guide (online supplemental appendix 4). The interview guide will be translated into isiXhosa, the local language. Interviews, expected to take an average of 45 min, will be held while patients wait for their appointments. Participants will be requested to participate in the study and to consent to an audio recording of the interviews. Audio recordings (online supplemental appendix 9) will be supplemented by field notes to serve as backup and to record observed expressions and gestures that participants will make. Participants who do not consent to an audio recording of their interviews will not be excluded but will only be recorded using field notes by a research assistant.

#### Substudy 2

In-person FGDs will be undertaken with healthcare providers at NMAH and SEH. Interview schedules (online supplemental appendix 5) will be used to ascertain the perceived burden of mental health illnesses associated with CC screening, diagnosis and management that health providers have observed in practice. Similar to the above substudy, consent for the audio recording of interviews will be sought from participants. FGDs will be in English and IsiXhosa and are expected to take an average of 30 min each. They will be held at a prearranged time with the health workers. Audio recordings will be supplemented by field notes to serve as a backup and record observed expressions and gestures that participants will make. Participants who do not consent to audio recording their FGD will not be excluded; they will only be recorded using field notes by a research assistant. In addition, an attendance register will be used to record participants’ job descriptions, ages and genders to summarise their demographic characteristics and to allow for a detailed description of participants’ diversity when sharing direct quotations. For both substudies 1 and 2, interview recordings will be transcribed verbatim, translated into English by a research assistant who was not involved in the transcription of that interview, and back translated into isiXhosa by a third research assistant who was not involved with the other two processes. This back translation will note errors that could have been introduced in the English translation and correct them to ensure consistency. Training will be provided for research assistants and interviewers.

#### Substudies 3 and 4

A combined total of 128 patients will have their quality of life assessed using the WHO-Quality of Life-shortened version (WHO-QoL-BREF) instrument (online supplemental appendix 6). This is a standardised 26-item tool that has shown good discriminant validity, content validity, internal consistency and test–retest reliability.^38 39^ This instrument is an in-person, self-administered questionnaire and assesses an individual’s perceptions of their health and well-being over the previous 2 weeks. It assesses four domains on a scale of 1–5: physical health, psychological well-being, social relationships and contextual factors in patients with multiple conditions. The WHO-QoL-BREF instrument will also include demographic and clinical characteristics, as detailed in items 1–6.

In addition, participants’ depression and anxiety will be screened using the PHQ-9 (online supplemental appendix 7) and the GAD-7 (online supplemental appendix 8) questionnaires, respectively.^40^ These instruments also screen for their conditions based on a patient’s reported feelings in the previous 2 weeks. The questionnaires will be administered in person by the researcher and trained research assistants. This face-to-face approach enables direct engagement, ensuring that participants feel at ease and can ask questions for clarification as needed. By delivering the materials personally, the study aims to foster a supportive environment that encourages honest and thoughtful responses.

The intervention will be the motivational interviewing (MI) of CC survivors by health promoters trained at Walter Sisulu University (WSU) with a Bachelor of Health Promotion. Participants in the hospital that will be randomised as a control site will all be handed psychoeducational handouts regardless of their screening outcomes.

### Training of health promoters in MI

All health promoters who will facilitate the motivational interviews will receive a 2-day MI training course using teaching manuals; the Motivational Interviewing Training New Trainers Manual; a pocket guide to MI^41^; and a participant’s manual on Substance Abuse and Mental Health Services Administration (SAMHSA).^42^ Fidelity of MI delivery will be assured through multiple complementary strategies. The principal investigator will oversee the delivery of MI, supervising the health promoters. Furthermore, all sessions will be recorded, and a checklist will be used to ensure that all topics are covered and that educational materials are provided. To enhance adherence to the mental wellness intervention, participants will receive regular SMS and phone call reminders before each health promoter-led session to support adherence. Attendance will be tracked, with follow-ups conducted within a week for those who miss sessions. Health promoters will offer motivational counselling and brief check-ins, while session logs will record engagement and barriers to ensure the intervention is delivered as intended.

### Intervention group

Patients in the intervention hospital will participate in four monthly face-to-face educational sessions (lasting 2 hours every month) over a consecutive 4-month period with the study participants, and they will also have peer sessions in between. The researcher (KS) will supervise the whole process.

Patients will be divided into four groups of 16 participants each. The sessions will share coping strategies for handling stress and social problems similar to those used by Myers *et al*.^37^ This substudy does not incorporate a process evaluation but instead seeks to test the possible effectiveness of this intervention and its feasibility in a low-resourced context. This intervention is a comprehensive, structured and person-centred counselling programme designed to support CC survivors on their journey towards improved health and well-being, led by trained health promoters. The content of the intervention will address the factors identified through both qualitative and quantitative studies. The study’s intended start date is 2 February 2026, and it is expected to conclude in May 2026.

### Data management and analysis

In-depth interviews and FGDs will be collected using a semistructured interview guide, audio recorded, transcribed verbatim and entered into NVivo V.15 for data analysis. Data will be analysed using a thematic analysis approach. Thematic analysis is a widely used qualitative data analysis method that involves identifying, analysing and reporting patterns (themes) within the data.^43^

In a quantitative study, data from baseline and postintervention outcome assessments will be captured electronically using a secure data management platform with built-in validation checks to ensure accuracy and minimise data entry errors. The data will then be exported to SPSS V.29 software (Statistical Package for the Social Sciences). The study will employ descriptive analyses of intervention participants’ responses, using means, SDs, medians and IQRs, as appropriate, based on the distribution’s normality. Outcome measures of participants in the intervention group will be compared with those in the control group using an appropriate t-test, χ^2^ test, Wilcoxon rank-sum test and/or the Fisher’s exact test, depending on the normality of the distribution and/or the value of the expected frequencies. The study will compare preinterview and postinterview guide summative scores using the paired t-test or the Wilcoxon signed-rank tests to ascertain patients’ mental health status and quality of life (comparing post-test with pretest scores), and whether mental health status and quality of life have changed after 4 months (comparing follow-up post-test with pretest scores). Univariable and multivariable analyses will also be undertaken using logistic regression to determine the effectiveness of health promoters on the primary outcome measures and factors that are associated with mental health risk among CC survivors. The risk ratio will be the relative measure of association used. The 95% CI will be used to assess the precision of estimates at the 5% level of significance (p≤0.05).

All analyses will follow the intention-to-treat principle. We will examine patterns of missing data to understand their underlying mechanisms. If the data are missing at random, we will use multiple imputation techniques to reduce potential bias. Additionally, sensitivity analyses will be performed to assess the robustness of our findings against various assumptions regarding the missing data. For secondary outcomes, we will apply the Bonferroni correction to adjust for multiple testing, ensuring that we maintain control over the overall type I error rate.

### Patient and public involvement

In this study, patients and the public will be actively involved to ensure the research is relevant, patient-centred and responsive to community needs across all substudies and phases.

Substudy 1: Patients diagnosed with CC will be engaged through interviews that explore their lived experiences, including the mental health impact linked to CC screening, diagnosis and treatment. Participants’ feedback will help identify key concerns related to psychological burden and quality of life, guiding the development of meaningful interview questions and the interpretation of findings. Participants will have the opportunity to comment on the study’s recommendations and dissemination strategies.

Substudy 2: Healthcare providers involved in CC screening and vaccination services will participate in focus groups to discuss barriers at both individual and systemic levels. Providers will also reflect on the mental health burdens they observe in patients, offering critical perspectives on care gaps. Their insights will contribute to refining interventions and improving service delivery models.

Phase II: The intervention developed from phase I findings will be codesigned with patient representatives and healthcare providers to ensure acceptability. Ongoing feedback mechanisms during the trial will support adjustments based on participant experiences.

The study will maintain transparent communication with patients and public representatives through regular updates, dissemination workshops and inclusion in advisory roles to strengthen the study’s impact and ethical conduct. The results will be published in peer-reviewed journals and presented at accredited conferences.

## Ethical and legal considerations and dissemination

The ethical clearance (online supplemental appendix 10) (protocol reference number: WSU HREC 023/2025) was approved by the WSU, Faculty of Medicine and Health Sciences, Ethics and Biosafety Committee. Access approval (online supplemental appendix 11) was obtained from the EC Provincial Health Research Committee (reference number: EC_202503_022). The OR Tambo District Office permitted the study within the OR Tambo District.

All participants will be provided with a written participant information sheet and informed consent form (online supplemental appendix 2) in the local language. The research staff at each site will be responsible for obtaining the participant information sheet and voluntary informed consent (online supplemental appendix 3). For participants who are unable to read or write, the participant information sheet will be read out to them, and the consent will be obtained using a thumbprint. Each consenting participant will be assigned a unique study identification number. To maintain confidentiality, all questionnaires and other study documents will have an identification number. Research records will be kept in a locked filing system until the completion of the study at WSU, and all electronic information will be securely stored in a password-protected computer and backed up to a password-encrypted storage cloud with two-factor authentication access. The data will be available and provided to the research team, including the principal investigator, research assistants, supervisors and the biostatistician, for the purposes of analysis and report writing. The study will abide by the four ethical principles of autonomy, non-maleficence, beneficence and justice. In this study, potential harms are described as emotional distress or psychological fatigue experienced by participants during mental wellness sessions. No physical harm is expected. Harms will be monitored through emotional check-ins, supervision meetings and participant reports. All concerns will be documented, reviewed and addressed following ethical guidelines for participant safety.

## Supplementary material

10.1136/bmjopen-2025-104827 online supplemental appendix 1

10.1136/bmjopen-2025-104827 online supplemental appendix 2

10.1136/bmjopen-2025-104827 online supplemental appendix 3

10.1136/bmjopen-2025-104827 online supplemental appendix 4

10.1136/bmjopen-2025-104827 online supplemental appendix 5

10.1136/bmjopen-2025-104827 online supplemental appendix 6

10.1136/bmjopen-2025-104827 online supplemental appendix 7

10.1136/bmjopen-2025-104827 online supplemental appendix 8

10.1136/bmjopen-2025-104827 online supplemental appendix 9

10.1136/bmjopen-2025-104827 online supplemental appendix 10

10.1136/bmjopen-2025-104827 online supplemental appendix 11
