# Supplementary material for: Protocol for exploring health promoter-led mental wellness initiatives for early prevention, screening and quality of life in patients with cervical cancer of rural Eastern Cape, South Africa: a mixed-methods study
Source: BMJ Open. 2026 Mar 25;16(3):e104827. doi: 10.1136/bmjopen-2025-104827 (PMC13034216; doi:10.1136/bmjopen-2025-104827)
Supplement: online supplemental appendix 1 [file bmjopen-16-3-s001.pdf]

SPIRIT 2025 checklist of items to address in a randomized trial protocol

Section / Topic	No	SPIRIT 2025 checklist item description	Reported on page no.
**Administrative information**			
Title and structured summary	1-3	A Protocol for Exploring Health Promoter-Led Mental Wellness Initiatives for Early Prevention, Screening, and Quality of Life in Cervical Cancer Patients of Rural Eastern Cape, South Africa: A mixed-method study	1
	262-263	Table 1: Research Methods Summary	10-11
Protocol version	14	Second version: October 2025	1
Roles and responsibilities	4-13	Khuthala Sigovana1,2, Sibusiso Nomatshila1,2, Wezile Chitha1,3, Sikhumbuzo A. Mabunda1,4,5,6<br>Walter Sisulu University, Public Health Department, Medical School, Mthatha, South Africa<br>1Public Health, Walter Sisulu University, Mthatha, South Africa<br>2Society and Health Research Institute, Walter Sisulu University, Mthatha, South Africa<br>3Institute for Clinical Governance and Healthcare Administration, Walter Sisulu University, Mthatha, South Africa<br>4Global Centre for Human Resources for Health Intelligence, Walter Sisulu University, Mthatha, South Africa<br>5School of Population Health, University of New South Wales, Sydney, Australia<br>6George Institute for Global Health, University of New South Wales, Sydney, Australia	1
	553	South African Medical Research Council	23
		The trial sponsor and funders will provide financial and logistical support but will have no role in the design, data collection, data analysis, interpretation of results, or writing of the final report. The study design, conduct, and reporting will remain under the full authority and independence of the principal investigator and the supervising university ethics committee. Any decisions to modify or terminate the study will be made solely by the principal investigator in consultation with the ethics committee, ensuring academic and scientific integrity throughout the research process.	
	499-504	Composition, roles, and responsibilities of the coordinating site, steering committee, endpoint adjudication committee, data management team, and other individuals or groups overseeing the trial, if applicable	21
**Open science**			
Trial registration	49	Pan African Clinical Trial Registry, identifying number (with URL), PACTR202507608317068, registered 24 June 2025.	2
Protocol and statistical analysis plan	469-497	In-depth interviews and Focus Group Discussions will be collected using a semi-structured interview guide, audio-recorded, transcribed verbatim, and entered into NVivo 15 for data analysis. Data will be analysed using a thematic analysis approach. Thematic analysis is a widely used qualitative data analysis method that involves identifying, analysing, and reporting patterns (themes) within the data (41).<br>In a quantitative study, data from baseline and post-intervention outcomes assessments will be captured electronically using a secure data management platform with built-in validation checks to ensure accuracy and minimize data entry errors. The data will then be exported to SPSS (Statistical	21

		Package for Social Sciences), software version 29. The study will employ descriptive analyses of intervention participants' responses, utilizing means, standard deviations, medians, and interquartile ranges, as appropriate, based on the distribution's normality. Outcome measures of participants in the intervention group will be compared with those in the control group using an appropriate t-test, chi-squared test, Wilcoxon sum-ranked test, and/or the Fisher's exact test, depending on the normality of the distribution, and or the value of the expected frequencies. The study will compare pre- and post-interview guide summative scores using the paired t-test or the Wilcoxon signed-rank tests to ascertain patients' mental health status and quality of life (comparing post-test with pre-test scores) and whether mental health status and quality of life have changed after four months (comparing follow-up post-test with pre-test scores). Univariable and multivariable analyses will also be undertaken using logistic regression to determine the effectiveness of health promoters on the primary outcome measures and factors that are associated with mental health risk among cervical cancer survivors. The risk ratio (RR) will be the relative measure of association used. The 95% confidence interval (95% CI) will be used to assess the precision of estimates at the 5% level of significance ($p \leq 0.05$).	
Data sharing	510-516	To maintain confidentiality, all questionnaires and other study documents will have an identification number. Research records will be kept in a locked filing system until the completion of the study at WSU, and all electronic information will be securely stored in a password-protected computer and backed up to a password-encrypted storage cloud with two-factor authentication access. The data will be available and provided to the research team, including the principal investigator, research assistants, supervisors, and the biostatistician, for the purposes of analysis and report writing.	21
Funding and conflicts of interest	551	Funding: This research received no external funding.	22
	552-553	The author(s) declare that they have no potential conflicts of interest concerning this protocol's research, authorship, and/or publication.	22
Dissemination policy	539-542	The study will maintain transparent communication with patients and public representatives through regular updates, dissemination workshops, and inclusion in advisory roles to strengthen the study's impact and ethical conduct. The results will be published in peer-reviewed journals and presented at accredited conferences. The trial will be reported to the trial registry as well.	22
**Introduction**			
Background and rationale	22-27	Cervical cancer is in the top five list of highly prevalent cancers. It contributes to millions of premature deaths globally. Low- and middle-income countries like those in sub-Saharan Africa are among the worst affected despite the preventable nature of the disease. The disease is caused by the human papillomavirus, a sexually transmitted infection for which an effective vaccine exists. This study aims to determine the effectiveness of a health promoter-led intervention on early screening, prevention, and quality of life improvement among patients with cervical cancer.	2
	255-259	Explanation for choice of comparator: The control group will continue to receive the usual care for cervical cancer treatment. At the end of the intervention period, the participants with cervical	10

		cancer in the control group will be revisited for outcome measures. They will further be referred to health facilities for definitive diagnosis and management of their mental health if at risk.	
Objectives	197-208	1) To explore the perceived burden of mental health illnesses associated with cervical cancer diagnosis, or treatment, targeting cervical cancer patients and healthcare workers in the OR Tambo district.<br>2) To assess barriers, practices, and knowledge on cervical cancer screening and HPV vaccination among women and health workers in the Eastern Cape.<br>3) To assess the quality of life of patients with a cervical cancer diagnosis and further determine factors affecting the patient's quality of life.<br>4) To analyse strategies for Health Promoter-Led Mental Wellness Initiatives for early screening and Quality of Life Improvement among Cervical Cancer Patients in the OR Tambo district.<br>5) To conduct a pre-post interventional evaluation of Mental Health Promoter-led early cervical cancer screening and treatment initiation.	8
**Methods: Patient and public involvement, trial design**			
Patient and public involvement	522-542	Patient and Public Involvement<br>In this study, patients and the public will be actively involved to ensure the research is relevant, patient-centered, and responsive to community needs across all sub-studies and phases.<br>Sub-study 1: Patients diagnosed with cervical cancer will be engaged through interviews that explore their lived experiences, including the mental health impact linked to cervical cancer screening, diagnosis, and treatment. Participants' feedback will help identify key concerns related to psychological burden and quality of life, guiding the development of meaningful interview questions and the interpretation of findings. Participants will have the opportunity to comment on the study's recommendations and dissemination strategies.<br>Sub-study 2: Healthcare providers involved in cervical cancer screening and vaccination services will participate in focus groups to discuss barriers at both individual and systemic levels. Providers will also reflect on the mental health burdens they observe in patients, offering critical perspectives on care gaps. Their insights will contribute to refining interventions and improving service delivery models.<br>Phase II: The intervention pilot developed from Phase I findings will be co-designed with patient representatives and healthcare providers to ensure acceptability and feasibility. Ongoing feedback mechanisms during the trial will support adjustments based on participant experiences.	21-22
Trial design	29-30 &221-222	The study will employ an exploratory sequential mixed-methods design, comprising four qualitative and quantitative sub-studies.	2,9
**Methods: Participants, interventions, and outcomes**			
Trial setting	212-218	The study will be conducted in Oliver Reginald Tambo district (Figure 1) within South Africa's Eastern Cape province (EC). This research will be conducted at the Nelson Mandela Academic Hospital, located in the King Sabata Dalindyebo subdistrict, and at the St Elizabeth Hospital, situated in the Ingquza Hill subdistrict.	8-9

Eligibility criteria	271-284	a) Sub-study 1: This will be in-depth interviews with 20 purposively sampled patients, 18 years and above, who have a cervical cancer diagnosis at both Nelson Mandela Academic Hospital (NMAH) and St. Elizabeth Hospital (SEH).<br>b) Sub-study 2: Healthcare workers, two to four focus group discussions will be held at NMAH and St. Elizabeth Hospital with nursing, medical, and allied health workers working in the two hospitals' oncology units.<br>c) Sub-study 3 and 4: Patients with a cervical cancer diagnosis at both NMAH and SEH hospitals will be requested to participate in a survey on their quality of life and their mental health status, specifically screening for depression and anxiety.	12
	435-436	The intervention will involve motivational interviewing of cervical cancer survivors by health promoters trained at Walter Sisulu University, who hold a Bachelor of Health Promotion degree.	18
Intervention and comparator	252-255 & 440-443	In the intervention group, four interventions will be implemented at one-month intervals, each followed by an evaluation of quality of life and mental health status outcomes. The four evaluations in the intervention group will run concurrently with those in the control group.<br>The intervention will be implemented by trained Health promoters utilising the Motivational Interviewing Training New Trainers Manual, along with a pocket guide for motivational interviewing and a participant's manual on substance abuse, during the intervention.	10,19
	N/A	Criteria for discontinuing or modifying allocated intervention/comparator for a trial participant (e.g., drug dose change in response to harms, participant request, or improving/worsening disease)	N/A
	447-551	To enhance adherence to the mental wellness intervention, participants will receive regular SMS and phone call reminders before each health promoter-led session to support adherence. Attendance will be tracked, with follow-ups conducted within a week for those who miss sessions. Health promoters will offer motivational counselling and brief check-ins, while session logs will record engagement and barriers to ensure the intervention is delivered as intended.	19
	255-269<br><br>364-367	The control group will continue to receive the usual care for cervical cancer treatment. At the end of the intervention period, the participants with cervical cancer in the control group will be revisited for outcome measures. They will further be referred to health facilities for definitive diagnosis and management of their mental health if at risk.<br><br>Referral pathways to psychiatric or crisis services are in place for those mental health patients who are excluded, ensuring they are not left without support. Notwithstanding, patients living with cervical cancer who have suicidal ideation may benefit from integrated mental health services, and their exclusion could delay access to care	10, 15
Outcomes	242-245<br><br>244-245	Primary outcomes: quality of life, and mental health indicators (anxiety and depression scores - GAD-7 and PHQ-9) will be evaluated in both groups as primary outcomes.<br>Secondary outcomes will include attendance rate, retention, and data completeness at intervention sessions.	9
Harms	516-521	The study will abide by the four ethical principles of Autonomy, Non-maleficence, Beneficence, and Justice. In this study, potential harms are described as emotional distress or psychological fatigue experienced by participants during mental wellness sessions. No physical harm is expected. Harms	21

		will be monitored through emotional check-ins, supervision meetings, and participant reports. All concerns will be documented, reviewed, and addressed following ethical guidelines for participant safety.	
Participant timeline	252-255	In the intervention group, four interventions will be implemented at one-month intervals, each followed by an evaluation of quality of life and mental health status outcomes. The four evaluations in the intervention group will run concurrently with those in the control group.	10
Sample size	286-299	The sample size was calculated using the equation, $n=\frac{(\sigma_1^2+\sigma_2^2)(Z_{1-\alpha}+Z_{1-\beta})^2}{(\mu_1-\mu_2)^2}$ for a two-sided 95% confidence interval, where ⍰ = 0.05, Z1-⍰ = 1.96, and for 80% power, Z1-⍰ = 0.84. The mean difference $\mu_1 - \mu_2 = 1$ and the standard deviation (σ) = 2. The sample size was calculated using a two-sample means formula, assuming a mean difference of 1 and a standard deviation of 2, which corresponds to a medium standardized effect size (Cohen's d = 0.5).	12-13
Recruitment	305-310	Participant recruitment will take place in collaboration with healthcare workers from the oncology units at Nelson Mandela Academic Hospital and St. Elizabeth Hospital. To raise awareness about the study and its benefits, informational sessions will be conducted within the hospitals, accompanied by posters displayed in key areas, such as the women's health units. Health promoters will actively engage potential participants, providing explanations about the study in local languages and assisting with the informed consent process.	13
**Methods: Assignment of interventions**			
Randomization:	236-238	A randomised control trial of 128 participants with a cervical cancer diagnosis from two hospitals Nelson Mandela Academic and St Elizabeth hospitals) will be recruited for the intervention.	9
Sequence generation	284-287	The sample size was calculated by the Biostatistician using the equation $n=\frac{(\sigma_1^2+\sigma_2^2)(Z_{1-\alpha}+Z_{1-\beta})^2}{(\mu_1-\mu_2)^2}$ for a two-sided 95% confidence interval, where $\alpha = 0.05$, $Z_{1-\beta} = 1.96$, and for 80% power, $Z_{1-\beta} = 0.84$. The mean difference $\mu_1 - \mu_2 = 1$ and the standard deviation (σ) = 2	12
	317-359	Cluster randomising at the hospital level (rather than individual level) will be employed to select patients with a cervical cancer diagnosis at both Nelson Mandela Academic and Saint Elizabeth hospitals to participate in a survey on their quality of life and their mental health status, specifically screening for depression and anxiety.	13-14
Allocation concealment mechanism	337-359	To ensure allocation concealment and minimise selection bias, the randomisation process will be managed independently by a designated biostatistician and data capturer who will not involved in participant recruitment or data collection. The random allocation sequence will be securely stored in a password-protected electronic system. The interventions and data collection will be conducted independently at each hospital. Participants will be randomly assigned to two groups, with 64 participants in each of the intervention and control groups.	14

Implementation	337-338	The personnel responsible for participant enrollment in this study will not have access to the random allocation sequence. Researchers will use an independent statistician and data manager to generate and hold the randomisation list.	14
Blinding	340-341	The outcome assessors will be blinded to group allocation to reduce potential bias in outcome measurement.	14
	337,354-355	Blinding will be achieved by ensuring that outcome assessors and data analysts remain unaware of group allocation throughout the data collection and analysis process. Limit the sharing of intervention materials or training across clusters until the study concludes.<br>The study will use separate WhatsApp groups, training sessions, and supervision structures for each arm.	14
	339	The allocation will be revealed only after the baseline data collection is complete.	14
**Methods: Data collection, management, and analysis**			
Data collection methods	419, 427-428, 441-443	A combined total of 128 patients will have their quality of life assessed using the WHO-QoL-BREF instrument (Appendix 7). In addition, participants' depression and anxiety will be screened using the PHQ-9 (Appendix 8) and the GAD-7 (Appendix 9) questionnaires, respectively. Health Promoters will utilise the Motivational Interviewing Training New Trainers Manual, along with a pocket guide for motivational interviewing and a participant's manual on substance abuse, during the intervention.	18,19
	447-451	To enhance adherence to the mental wellness intervention, participants will receive regular SMS and phone call reminders before each health promoter-led session to support adherence. Attendance will be tracked, with follow-ups conducted within a week for those who miss sessions. Health promoters will offer motivational counselling and brief check-ins, while session logs will record engagement and barriers to ensure the intervention is delivered as intended.	19
Data management	474-477	Data from baseline and post-intervention outcomes assessments will be captured electronically using a secure data management platform with built-in validation checks to ensure accuracy and minimize data entry errors. The data will then be exported to SPSS (Statistical Package for Social Sciences), software version 29	20
Statistical methods	478-497	The study will employ descriptive analyses of intervention participants' responses, utilizing means, standard deviations, medians, and interquartile ranges, as appropriate, based on the distribution's normality. Outcome measures of participants in the intervention group will be compared with those in the control group using an appropriate t-test, chi-squared test, Wilcoxon sum-ranked test, and/or the Fisher's exact test, depending on the normality of the distribution, and or the value of the expected frequencies. The study will compare pre- and post-interview guide summative scores using the paired t-test or the Wilcoxon signed-rank tests to ascertain patients' mental health status and quality of life (comparing post-test with pre-test scores) and whether mental health status and quality of life have changed after four months (comparing follow-up post-test with pre-test scores). Univariable and multivariable analyses will also be undertaken using logistic regression to determine the effectiveness of health promoters on the primary outcome measures and factors that are associated with mental health risk among cervical cancer survivors. The risk ratio (RR) will	20-21

		be the relative measure of association used. The 95% confidence interval (95% CI) will be used to assess the precision of estimates at the 5% level of significance ($p \leq 0.05$).	
	337-338	Researchers will use an independent biostatistician and data capturer/manager to generate and maintain the randomisation list in both the intervention and control groups.	14
	475-476	Data from baseline and post-intervention outcomes assessments will be captured electronically using a secure data management platform with built-in validation checks to ensure accuracy and minimize data entry errors.	20
	492-497	Methods for any additional analyses (e.g., subgroup and sensitivity analyses)	17-18, 20-21
**Methods: Monitoring**			
Data monitoring committee	501-505& 521-524	The ethical clearance (Appendix 10) with protocol reference number WSU HREC 023/2025 was approved by the Walter Sisulu University, Faculty of Medicine and Health Sciences Ethics and Biosafety Committee. Access approval (Appendix 11) was obtained from the Eastern Cape Provincial Health Research Committee (reference number EC_202503_022). The OR Tambo district's office permitted the study in the OR Tambo district. Ongoing monitoring of participant well-being and data quality will be conducted throughout the research period. The principal investigator, together with the university ethics committee and project supervisors, will review any safety or ethical concerns that may arise. The trial may be terminated early if there is evidence of participant harm, ethical non-compliance, or circumstances that compromise data integrity or participant welfare. The principal investigator and ethics committee will have access to any interim safety summaries and will make the final decision to continue, modify, or terminate the study.	21
	501-505& 521-524	No formal interim analyses will be done, but participant well-being and data quality will be continuously monitored. The principal investigator, ethics committee, and supervisors will review safety or ethical issues. The trial may be terminated early if harm, ethical breaches, or data issues occur. Only the principal investigator and the ethics committee can access interim safety data and decide whether to continue or terminate the study.	20,21
Trial monitoring	501-505, 521-524, 542-545	Frequency and procedures for monitoring trial conduct. If there is no monitoring, give explanation	21,22
**Ethics**			
Research ethics approval	501-505	The ethical clearance (Appendix 11) with protocol reference number WSU HREC 023/2025 was approved by the Walter Sisulu University, Faculty of Medicine and Health Sciences Ethics and Biosafety Committee. Access approval (Appendix 12) was obtained from the Eastern Cape Provincial Health Research Committee (reference number EC_202503_022).	21
Protocol amendments	501-505<br><br>542-545	Any substantive protocol modifications will be submitted for prior review and approval by the Walter Sisulu University, Faculty of Medicine and Health Sciences Ethics and Biosafety Committee and the Eastern Cape Provincial Health Research Committee before implementation. Approved amendments will be documented with version control and communicated to all relevant study personnel and sites. Participants will be informed of any changes that may influence their participation or consent.	21,22

Consent or assent	506-507	The research staff at each site will be responsible for obtaining voluntary informed consent form.	21
		N/A	16
Confidentiality	510-518	To maintain confidentiality, all questionnaires and other study documents will have an identification number. Research records will be kept in a locked filing system until the completion of the study at WSU, and all electronic information will be securely stored in a password-protected computer and backed up to a password-encrypted storage cloud with two-factor authentication access. The data will be available and provided to the research team, including the principal investigator, research assistants, supervisors, and the biostatistician, for the purposes of analysis and report writing.	21
Ancillary and post-trial care	364 -365 & 529-530	Provisions, if any, for ancillary and post-trial care, and for compensation to those who suffer harm from trial participation	15,22
