# Supplementary material for: Protocol for exploring health promoter-led mental wellness initiatives for early prevention, screening and quality of life in patients with cervical cancer of rural Eastern Cape, South Africa: a mixed-methods study
Source: BMJ Open. 2026 Mar 25;16(3):e104827. doi: 10.1136/bmjopen-2025-104827 (PMC13034216; doi:10.1136/bmjopen-2025-104827)
Supplement: online supplemental appendix 3 [file bmjopen-16-3-s003.pdf]

Appendix 3: Information sheet and consent form for healthcare workers (English version)

I am (Name of the Researcher) ……………………a PhD student/researcher in the Department of Public Health at Walter Sisulu University (WSU). My research topic is: Health Promoter-Led Mental Wellness Initiatives for Early Prevention, Screening, and Quality of Life in Cervical Cancer Patients of Rural Eastern Cape, South Africa**.**

The research is conducted as a requirement of the degree program, which will lead to my completion of the Doctor of Philosophy in Health Sciences. You are therefore invited to consider participating in this research.

Expectations by the participants: Participation in this study requires obtaining your consent to participate voluntarily, and you may withdraw at any point without incurring any penalty. If you choose to participate, your participation is expected to be 30 - 45 minutes for the interview session. Your confidentiality will be maintained throughout your participation, and your name is not required on the data collection tools to enhance anonymity.

Safety of the study: There is no risk involved in your participation, as no invasive procedure will be used to collect data. This study involves participants sharing insight on investigating the burden of mental health illnesses associated with cervical cancer screening, diagnosis, or treatment.

In the event of any concerns, you may contact the research supervisor:

Prof. …………………………….. at WSU, Department of Public Health.

Email:

My contact details are:

Researcher: ………………………………

Cell Number:

Email:

Consent Form

I ………………………………………….. (participant name) have been informed about the study entitled: ………………………………………………………………………………

I understand the purpose and procedures of the study. I have been allowed to answer questions about the research and have had answers to my satisfaction. I declare that my participation in this study is entirely voluntary.

Audio Recording Consent

The interview will be audio recorded, and the recorded tape will be disposed of after the interview has been transcribed and the information has been compiled into a report.

Statement of Consent to be Audio taped

I understand that audio recordings will be taken during the study. (Tick either "Yes" or "No")

I agree to being audio-recorded

Yes	
No	

______________________ ______________________

Signature of Participant **Date**

Signature of researcher

I believe the participant is giving informed consent to participate in this study.

________________________ _______________________

Signature **Date**
