# Supplementary material for: Protocol for exploring health promoter-led mental wellness initiatives for early prevention, screening and quality of life in patients with cervical cancer of rural Eastern Cape, South Africa: a mixed-methods study
Source: BMJ Open. 2026 Mar 25;16(3):e104827. doi: 10.1136/bmjopen-2025-104827 (PMC13034216; doi:10.1136/bmjopen-2025-104827)
Supplement: online supplemental appendix 4 [file bmjopen-16-3-s004.pdf]

Appendix 4: Interview guide (English version)

Semi-structured Interview Guide

Duration: 30 to 45 minutes

Health Promoter-Led Mental Wellness Initiatives for Early Prevention, Screening, and Quality of Life in Cervical Cancer Patients of Rural Eastern Cape, South Africa

Participants: Cervical Cancer Survivors

Questions

1. **Tell me about your age and place of residence stay.**

Answer:
……………………………………………………………………………………………………
…………………………………………………………………………………………………..

2. **Can you please tell me more about when you were diagnosed with cervical cancer? Which year was it? Were you employed? Were you married? Did you have Children? Who were you staying with?**

Answer:

……………………………………………………………………………………………………
…………………………………………………………………………………………………..

3. **Please tell me what you know about mental health illnesses.**

Answer……………………………………………………………………………………………
……………………………………………………………………………………………………

Probing questions:

3.1 Please tell me more.

Answer:
……………………………………………………………………………………………………
………………………………………………………………………………………………….

4. **What is the burden of mental health illnesses associated with cervical cancer screening, diagnosis, and treatment?**

Answer:

……
………..

Probing questions on screening:

4.1 How did you feel as a patient when you were offered cervical cancer screening?

………

4.2 Did you have any specific fears or anxieties related to cervical cancer screening?

……….

Please tell me more.

4.3 How did you feel about the information you received during the screening process?

……………………………………………………………………………………….

5. **What mental health challenges have you experienced after your cervical cancer diagnosis?**

Answer:

……
………..

Probing questions:

5.1 Are there any specific mental symptoms or illnesses you have experienced or any other conditions like HIV, Hypertension, or diabetes?

………

5.2 Please tell me more…………………………………………………………………………

5.3 How did the prognosis impact your mental health outcomes?

……….

5.4 What mental health challenges have you experienced soon after your diagnosis compared to now?

Answer:

……

6. What mental health challenges have you experienced when undergoing different types of cervical cancer treatment, such as surgery, chemotherapy, or radiation?

Answer

…….

Probing questions:

6.1 How did these side effects from the treatment affect/impact your mental state?

Answer:

……………………………………………………………………………………..

Probing questions:

6.2 What is the overall experience of cervical cancer care impacting on your mental health?

………………………………………………………………………………………………………..

Answer:

………………………………………………………………………………………………………..

Thank you. We have come to the end of the interview. Do you have any questions for me?
