# Supplementary material for: Protocol for exploring health promoter-led mental wellness initiatives for early prevention, screening and quality of life in patients with cervical cancer of rural Eastern Cape, South Africa: a mixed-methods study
Source: BMJ Open. 2026 Mar 25;16(3):e104827. doi: 10.1136/bmjopen-2025-104827 (PMC13034216; doi:10.1136/bmjopen-2025-104827)
Supplement: online supplemental appendix 5 [file bmjopen-16-3-s005.pdf]

**Appendix 5: Interview guide (English version)**

**Interview Guide**

**Duration: 30 to 45 minutes**

**Health Promoter-Led Mental Wellness Initiatives for Early Prevention, screening, and Quality of Life in Cervical Cancer Patients of Rural Eastern Cape, South Africa.**

**Participants: Healthcare workers**

**Questions**

1. **Tell me about your occupation**

Answer:
……………………………………………………………………………………………………

2. **Identification of individual and system-level barriers to cervical cancer screening and vaccination**

2.1 In your experience or view, how much do patients know about cervical cancer screening and vaccination?

Answer:

……………………………………………………………………………………………

Probing question: Are there any misconceptions/beliefs about cervical screening and vaccination?

………………………………………………………………………………………………

2.2 What individual barriers do you think might be the hindrances to early cervical screening?

Answer:

………………………………………………………………………………………………

2.3 What barriers do you see at the facility level that hinder cervical cancer screening services?

Answer:

………………………………………………………………………………………………

2.4 Do you think your facility has enough resources for effective cervical screening services?

Answer:

…………………………………………………………………………………………………

2.4.1 Tell me more about these resources:

Answer:

…………………………………………………………………………………………………

2.5 Is there any form of training offered to healthcare workers on pap smears?

Answer:

…………………………………………………………………………………………………

Probing questions:

2.5.1 Tell me more.

3. **Can you please tell me what you know about mental health illnesses from cervical cancer patients?**

Answer:

…………………………………………………………………………………………………

Probing questions:

3.1 Please tell me more.

………………………………………………………………..

4. **What is the burden of mental health illnesses associated with cervical cancer screening, diagnosis, and treatment?**

Answer:

……………………………………………………………………………………………………

Probing questions on screening:

4.1 How do patients feel when they are offered cervical cancer screening?

……………………………………………………………………………………………………

4.2 What signs do they have to show specific fears or anxieties related to cervical cancer screening?

……………………………………………………………………………………………………

Please tell me more.

3.3 How do patients feel about the information they receive during the screening process?

…………………………………………………………………………………………………….

5. **What mental health challenges are reported the most by cervical cancer patients after cervical cancer diagnosis?**

Answer:

………………………………………………………………………………………………………

Probing questions:

5.1 Are there any specific mental symptoms or illnesses that are reported?

……………………………………………………………………………………………………

5.2 Please tell me more…………………………………………………………………………

5.3 How does the prognosis impact the mental health outcomes of the patients?

………………………………………………………………………………………………………

5.4 Do patients report any differences in mental health burdens/illnesses associated with early-stage and advanced-stage diagnosis?

Answer:

………………………………………………………………………………………………………

6. **Are there any mental health challenges reported by patients undergoing different types of cervical cancer treatment, such as surgery, chemotherapy, or radiation?**

Answer

……………………………………………………………………………………………………

Probing questions:

6.1 How do side effects from the treatment affect/impact a patient's mental state?

Answer:

………………………………………………………………………………………………………

Probing questions:

6.2 What is the overall experience of cervical cancer care impacting mental health?

…………………………………………………………………………………………

Answer:

…………………………………………………………………………………………

**Thank you. We have come to the end of the interview. Do you have any questions for me?**
