# Supplementary material for: Protocol for exploring health promoter-led mental wellness initiatives for early prevention, screening and quality of life in patients with cervical cancer of rural Eastern Cape, South Africa: a mixed-methods study
Source: BMJ Open. 2026 Mar 25;16(3):e104827. doi: 10.1136/bmjopen-2025-104827 (PMC13034216; doi:10.1136/bmjopen-2025-104827)
Supplement: online supplemental appendix 6 [file bmjopen-16-3-s006.pdf]

Appendix 6: WHO Quality of Life Scale-Brief English version

Before we begin, we would like to ask you to answer a few general questions about yourself by circling the correct answer or filling in the space provided.

1. What is your gender? Male Female
2. What is your date of birth? ________ / ________ / ________
   Day Month Year
3. What is the highest education you received? None at all □ Primary school □ High School □ College/University □ Graduate/Professional□
4. What is your marital status? Single □ Married□ Separated□ Divorced□ Living as Married□ Widowed□ Cohabiting□
5. Are you currently ill? Yes No
6. If something is wrong with your health, what illness/problem do you think it is? __________________

Instructions: This questionnaire asks how you feel about your quality of life, health, or other areas of your life. Please answer all of the questions. If you are unsure about which response to give to a question, please choose the one that appears most appropriate. This can often be your first response.

Please keep in mind standards, hopes, pleasures, and concerns. We ask that you think about your life in the last two weeks. For example, thinking about the previous two weeks, a question might ask:

Do you get the kind of support that you need from others?

(Please circle the number)				
Not at all<br>1	A little<br>2	Moderately<br>3	Mostly<br>4	Completely<br>5

You should circle the number that best fits how much support you got from others over the last two weeks. So you would circle the number 4 if you got much support from others.

Do you get the kind of support that you need from others?

(Please circle the number)				
Not at all	A little	Moderately	Mostly	Completely
1	2	3	4	5

You would circle number 1 if you did not get any of the support that you needed from others in the last two weeks.

Do you get the kind of support from others that you need?

(Please circle the number)				
Not at all	A little	Moderately	Mostly	Completely
1	2	3	4	5

Please read each question, assess your feelings, and circle the number on the scale that gives the best answer for you for each question.

1. How would you rate your quality of life?

(Please circle the number)				
Very poor 1	Poor 2	Neither poor nor good 3	Good 4	Very Good 5

2. How satisfied are you with your health?

(Please circle the number)				
Very dissatisfied 1	Dissatisfied 2	Neither satisfied nor dissatisfied 3	Satisfied 4	Very satisfied 5

The following questions ask about **how much** you have experienced certain things in the last two weeks.

(Please circle the number)				
Not at all	A little	A moderate amount	Very much	An extreme amount
1	2	3	4	5

3. To what extent do you feel that physical pain prevents you from doing what you need to do? **1 2 3 4 5**

4. How much do you need any medical treatment to function in your life? **1 2 3 4 5**

5. How much do you enjoy life? **1 2 3 4 5**

6. To what extent do you feel your life to be meaningful? **1 2 3 4 5**

7. How well are you able to concentrate? **1** **2** **3** **4** **5**

(Please circle the number)				
Not at all 1	Slightly 2	A Moderate amount 3	Very Much 4	Extremely 5

8. How safe do you feel in your daily life? 1 2 3 4 5

9. How healthy is your physical environment? 1 2 3 4 5

The following questions ask about **how completely** you experience or were able to do certain things in the last two weeks.

(Please circle the number)				
Not at all 1	A little 2	Moderately 3	Mostly 4	Completely 5

10. Do you have enough energy for everyday life? 1 2 3 4 5

11. Are you able to accept your bodily appearance? 1 2 3 4 5

12. Have you enough money to meet your needs? **1** **2** **3** **4** **5**

13. How available to you is the information that you need in your day-to-day life? **1** **2** **3** **4** **5**

14. To what extent do you have the opportunity for leisure activities? **1** **2** **3** **4** **5**

		(Please circle the number)		
Very poor 1	Poor 2	Neither poor nor well 3	Well 4	Very well 5

15. How well are you able to get around?

The following questions ask you to say how **good** or **satisfied** you have felt about various aspects of your life over the last two weeks.

(Please circle the number)				
Very dissatisfied 1	Dissatisfied 2	Neither satisfied nor dissatisfied 3	Satisfied 4	Very satisfied 5

16. How satisfied are you with your sleep?

17. How satisfied are you with your ability to perform your daily living activities? **1 2 3 4 5**

	(Please circle the number)			
Very dissatisfied 1	Dissatisfied 2	Neither satisfied nor dissatisfied 3	Satisfied 4	Very satisfied 5

18. How satisfied are you with your capacity for work? **1 2 3 4 5**

19. How satisfied are you with yourself? **1 2 3 4 5**

20. How satisfied are you with your personal relationships?**1 2 3 4 5**

21. How satisfied are you with your sex life? **1 2 3 4 5**

22. How satisfied are you with the support you get from your friends? **1 2 3 4 5**

23. How satisfied are you with the conditions of your living place?**1 2 3 4 5**

24. How satisfied are you with your access to health services? **1 2 3 4 5**

25. How satisfied are you with your mode of transportation?**1 2 3 4 5**

The following question refers to **how often** you have felt or experienced certain things in the last two weeks.

	(Please circle the number)				
	Never 1	Seldom 2	Quite often 3	Very often 4	Always 5
**26.** How often do you have negative feelings, such as blue mood, despair, anxiety, or depression?	**1**	**2**	**3**	**4**	**5**

(Please circle Yes or No

Did someone help you to fill out this form? Yes No

How long did it take you to fill out this form? _______________minutes
