# Supplementary material for: Protocol for exploring health promoter-led mental wellness initiatives for early prevention, screening and quality of life in patients with cervical cancer of rural Eastern Cape, South Africa: a mixed-methods study
Source: BMJ Open. 2026 Mar 25;16(3):e104827. doi: 10.1136/bmjopen-2025-104827 (PMC13034216; doi:10.1136/bmjopen-2025-104827)
Supplement: online supplemental appendix 7 [file bmjopen-16-3-s007.pdf]

Appendix 7: PATIENT HEALTH QUESTIONNAIRE (PHQ-9) English version

ID#____ DATE:______________________

Over the last two weeks, how often have you been bothered by any of the following problems?

Use (✓) to indicate your answer

	Not al all	Several days	More than half the days	Nearly every day
1. Little interest or pleasure in doing things	0	1	2	3
2. Feeling down, depressed, or hopeless				
3. Trouble falling or staying asleep or sleeping too much				
4. Feeling tired or having little energy				
5. Poor appetite or overeating				
6. Feeling bad about yourself-or that you are a failure or have let yourself or your family down.				
7. Trouble concentrating on things, such as reading the newspaper or watching television.				
8. Moving or speaking so slowly that other people could have noticed. Or the opposite-being, so fidgety or restless that you have been moving around a lot more than usual				
9. Thoughts that you would be better off				

dead, or hurting yourself.				
	Add columns	+	+	
	TOTAL:			

(*Health care professional: For interpretation of TOTAL,*

please refer to accompanying scoring card)

If you checked *off any problems*, how *difficult* have these problems made for you to do your work, take care of things at home, or get along with other people?	No difficult at all ______________<br>Somewhat difficult ______________<br>Very difficult ______________<br>Extremely difficult ______________

Interpretation of Total Score

Total Score	Depression Severity
1-4	Minimal depression
5-9	Mild depression
10-14	Moderate depression
15-19	Moderately severe depression
20-27	Severe depression

PHQ9 Copyright © Pfizer Inc. All rights reserved. Reproduced with permission. PRIME-MD ® is a trademark of Pfizer Inc.
