# Supplementary material for: Protocol for exploring health promoter-led mental wellness initiatives for early prevention, screening and quality of life in patients with cervical cancer of rural Eastern Cape, South Africa: a mixed-methods study
Source: BMJ Open. 2026 Mar 25;16(3):e104827. doi: 10.1136/bmjopen-2025-104827 (PMC13034216; doi:10.1136/bmjopen-2025-104827)
Supplement: online supplemental appendix 9 [file bmjopen-16-3-s009.pdf]

Appendix 9: Qualitative data collection checklist

Audio file name:

Items	Option	Answer
Sex	Female/Male	
Respondent Category	DOH District Staff/ patients	
Province		
**Ethics Checklist**	**Yes**	**No**
Have you explained the purpose of the research?		
Have you explained issues related to confidentiality?		
Have you explained how long the interview will take?		
Have you obtained informed consent?		
Have you explained the preferred language of the interview?		
Can the interview be recorded?		
If No, can detailed notes be taken?		
Do you have any questions before the recording starts?		
