# Supplementary material for: Protocol for exploring health promoter-led mental wellness initiatives for early prevention, screening and quality of life in patients with cervical cancer of rural Eastern Cape, South Africa: a mixed-methods study
Source: BMJ Open. 2026 Mar 25;16(3):e104827. doi: 10.1136/bmjopen-2025-104827 (PMC13034216; doi:10.1136/bmjopen-2025-104827)
Supplement: online supplemental appendix 10 [file bmjopen-16-3-s010.pdf]

WSU Walter Sisulu University
In pursuit of excellence

WALTER SISULU UNIVERSITY HEALTH SCIENCES RESEARCH ETHICS COMMITTEE
(NHREC Reg. number REC-120209-020)

**Ethics Approval Number**	WSU HREC 023/2025
**Project Title**	Health Promoter-Led Mental Wellness Initiatives for early prevention, screening, and quality of life in cervical cancer patients of rural Eastern Cape, South Africa
**Principal Researcher/ Investigator**	Ms Khuthala Sigovana
**Supervisor/s**	Prof SA Mabunda **Co-supervisors:** Prof WW Chitha & Prof SC Nomatshila
**Collaborator/s**	N/A
**Department**	Public Health
**Faculty**	Medicine & Health Sciences
**Type of Risk**	None
**Nature of the Project**	Undergrad [ ] Hons [ ] Masters [ ] Doctoral [X] Departmental [ ] Clinical Trial [ ] External Researcher [ ]
**Ethics Approval Commencement Date**	20 March 2025 — **Expiry Date** 19 March 2026

Walter Sisulu University Health Research Ethics Committee (WSU HREC) hereby grants ethical approval for 1 year in respect of the undertakings submitted by the research project mentioned above. The researcher needs to submit to the HREC the gatekeeper/s' approval letters and certificate of registration of the study with the National Health Research Database before data collection.

Conditions:

i. The certificate is valid for 1 year.
ii. The Principal Investigator/s is/ are required to provide the committee with a progress or outcome report of the research after every 1 year, 1 month before the expiry of the ethics clearance and the due date is **19 February 2026.**
iii. Please submit study closure report should the study be completed within approval period as final submission; and if the study continues beyond approval, please submit a progress report and request for extension of ethics clearance.
iv. The approval is strictly for the submitted proposal, any changes/amendments in the protocol must first be submitted and approved by the HREC as well as any adverse events or deviations that may occur at any time during the study must be submitted not later than 7 days of knowing as the investigator/s.
v. The committee expects a report on any changes in the protocol as well as any untoward events that may occur at any time during the study not later than 7 days of knowing as the investigator/s.
vi. As indicated in the Protection of Personal Information Act 04 of 2013 (POPIA), researchers have a legal duty to protect participants' personal information that they process. They must ensure privacy and security for all personal information process through research. They need to explicitly define the lawful purpose of collecting personal information and the duration that they are going to keep it. Collected personal information must be de-identified and destroyed when it is no longer in use. Participants should always give their consent and be informed that should they decide to leave the research they will not be disadvantaged at any point.
vii. **The investigator must submit a copy of the Site/ Gatekeeper permission to the HREC before commencing data collection.**
viii. The HREC must be informed immediately of discontinuation of the study and state the reasons.

The WSU HREC wishes you well with your study.

Walter Sisulu University
Health Research Ethics Committee
24 MAR 2025
Faculty of Medicine
and Health Sciences

....
PROF EJ NDEBIA
WSU HREC Chairperson
Date: 20 MARCH 2025

DECLARATION OF INVESTIGATOR(S)

(To be completed in duplicate and one copy returned to the Research Officer at Office AB 02 GF 03 Administration Building, Sisson Street Campus, Fort Gale, Mthatha, WSU)

I/We fully understand the conditions under which I am/we are authorized to carry out the abovementioned research and I/we guarantee to ensure compliance with these conditions. Should any departure to be contemplated from the research procedure as approved I/we undertake to resubmit the protocol to the WSU Research Ethics Committee. I/We agree to a completion of a yearly progress/ final report. The committee reserves the right to withdraw approval in the event that there are serious ethical violations.

.... (Signature) 24/03/2025 .... (Date)

N. B. Please quote the protocol number in all enquiries.
