# Supplementary material for: Protocol for exploring health promoter-led mental wellness initiatives for early prevention, screening and quality of life in patients with cervical cancer of rural Eastern Cape, South Africa: a mixed-methods study
Source: BMJ Open. 2026 Mar 25;16(3):e104827. doi: 10.1136/bmjopen-2025-104827 (PMC13034216; doi:10.1136/bmjopen-2025-104827)
Supplement: online supplemental appendix 11 [file bmjopen-16-3-s011.pdf]

Province of the
EASTERN CAPE
HEALTH

Room 31 • 1st Floor • Grosvenor Lodge • 31 Taylor Street • King Williams Town• Eastern Cape
Private Bag X0038 • Bhisho • 5605 • REPUBLIC OF SOUTH AFRICA
Tel.: +27 (0)43 605 4535• 043 6054518 •Email: ncebagixela22@gmail.com

Date: 31 March 2025

Health Promoter-Led Mental Wellness Initiatives for early prevention, screening, and quality of life in cervical cancer patients of rural Eastern Cape, South Africa. (EC_202503_022)

Dear Ms. Khuthala Sigovana

The department would like to inform you that your application for the research topic mentioned above has been approved based on the following conditions:

1. During your study, you will follow the submitted protocol with ethical approval and can only deviate from it after having written approval from the Research Ethics Committee.

2. You are advised to ensure, observe, and respect the rights and culture of your research participants, maintain confidentiality of their identities, and remove or not collect any information that can be used to link the participants.

3. The Department of Health expects you to provide a progress update on your study every 3 months (from the date you received this letter) in writing.

4. At the end of your study, you will be expected to send a full written report with your findings and implementable recommendations to the Eastern Cape Health Research Committee secretariat. You may also be invited to the department to come and present your research findings with your implementable recommendations.

5. Your results on the Eastern Cape will not be presented anywhere unless you have shared them with the Department of Health as indicated above.

Your compliance in this regard will be highly appreciated.

GN. Gixela 31.03.2025

SECRETARIAT: EASTERN CAPE HEALTH RESEARCH COMMITTEE
