# Supplementary material for: Protocol for exploring health promoter-led mental wellness initiatives for early prevention, screening and quality of life in patients with cervical cancer of rural Eastern Cape, South Africa: a mixed-methods study
Source: BMJ Open. 2026 Mar 25;16(3):e104827. doi: 10.1136/bmjopen-2025-104827 (PMC13034216; doi:10.1136/bmjopen-2025-104827)
Supplement: Supplementary file 12 [file bmjopen-2025-104827.reviewer_comments.pdf]

PEER REVIEW HISTORY

BMJ Open publishes all reviews undertaken for accepted manuscripts. Reviewers are asked to complete a checklist review form and are provided with free text boxes to elaborate on their assessment. These free text comments are reproduced below.

ARTICLE DETAILS

Title (Provisional)

A Protocol for Exploring Health Promoter-Led Mental Wellness Initiatives for Early Prevention, Screening, and Quality of Life in Cervical Cancer Patients of Rural Eastern Cape, South Africa: A mixed-method study

Authors

Sigovana, Khuthala; Nomatshila, Sibusiso C.; Chitha, Wezile; Mabunda, Sikhumbuzo A.

VERSION 1 - REVIEW

**Reviewer**	**1**
**Name**	**Cwik, Jan**
**Affiliation**	**Universität zu Köln, Clinical Psychology and Psychotherapy**
**Date**	**12-Sep-2025**
**COI**	**None**

This study protocol outlines an exploratory sequential mixed-method design to evaluate the effectiveness and feasibility of a health promoter-led intervention aimed at improving mental wellness, early screening, and quality of life in cervical cancer patients in the OR Tambo district, South Africa. The protocol includes qualitative in-depth interviews and focus groups (Phase I) and a small randomized feasibility trial (Phase II) with pre- and post-intervention assessments using validated instruments (WHOQoL-BREF, PHQ-9, GAD-7).

• The topic is timely and highly relevant in a low-resource setting with high cervical cancer incidence and mortality.

• The mixed-method approach is appropriate first to explore barriers and inform the design of the intervention before testing feasibility and preliminary effectiveness.

• The use of validated mental health and quality-of-life instruments is a strong methodological choice, as it facilitates comparability with other research.

• The inclusion of health promoters and community engagement throughout the study enhances the ecological validity and potential for sustainability.

However, some methodological aspects could be improved or clarified:

• Although a power calculation is provided for the quantitative phase, it would be helpful to clarify the anticipated effect size and provide justification for its choice. As this is a feasibility study, the authors could also state explicitly that the primary aim is to assess feasibility parameters (e.g., recruitment, retention, adherence) rather than to achieve statistical power for hypothesis testing.

• More details are needed on the randomization process—specifically, how cluster randomization at the hospital level will be implemented, whether allocation concealment will be used, and how potential contamination between groups will be minimized.

• The protocol does not mention whether outcome assessors will be blinded to group allocation. This should be addressed to reduce potential bias in outcome measurement.

• While descriptive and inferential analyses are described, it would be valuable to specify how missing data will be handled and whether adjustments for multiple testing will be considered for secondary outcomes.

• The description of the training for health promoters is clear; however, the protocol should also specify how the fidelity of motivational interviewing delivery will be monitored (e.g., supervision, checklists, session recordings).

• The intervention lasts four months, with no planned long-term follow-up. This should be explicitly stated as a limitation, given that the sustainability of mental health improvements cannot be assessed.

• The protocol outlines clear procedures for obtaining informed consent and protecting data. However, given that participants with significant mental health issues (e.g., suicidal ideation) will be excluded, the authors could discuss the ethical and practical implications of excluding a potentially vulnerable subgroup.

• Since this is a feasibility study, it would be useful to pre-specify key feasibility outcomes (e.g., recruitment rate, session attendance, retention) and success criteria for proceeding to a full-scale trial.

Overall, this protocol is well written, addresses a significant public health challenge, and is aligned with BMJ Open's mission to publish high-quality, transparent study protocols. By clarifying the randomization and analysis plan, specifying feasibility outcomes, and describing procedures to ensure intervention fidelity, this study will make a valuable contribution to evidence on community-based mental health interventions in cancer care in low-resource settings. Its focus on health promoter-led models and integration of mental health with cancer care make it particularly relevant for global health and implementation science audiences.

Reviewer 2

Name **Garrett, Nick**

Affiliation **AUT University, Department of Biostatistics and Epidemiology**

Date **22-Sep-2025**

COI **None**

The article is generally well written, however there is some inconsistencies and lack of details in some areas:

- There is a lack of clarity as to how the quantitative study participants are going to be recruited. Additionally there is a reference to outpatients in the abstract and data management section but not referred to elsewhere.

Background

- page 4 lines 73-74, a statement is made about the increasing incidence and mortality in Africa, however it is not referenced. later in the page on lines 80-82 the statement is re-stated and this time has a reference. I recommend that the first statement is removed.

- page 5 line 97 there is a statement that women living with HIV have an earlier onset of CC, I am unable to access the reference, but given risk factors for CC and HIV have some overlap it is not clear whether women living with HIV had the onset of CC while living with HIV or potentially prior to living with HIV?

Objectives

- given that all participants (quantitative and qualitative) that are not clinical staff are women who have been diagnosed with CC, I am wary of including mental health status associated with CC screening in objective 1, unless the objective is slightly re-framed.

Methods

- The qualitative Phase I appears to be well designed,

- However Phase II lacks some clarity, there is reference to 2 sub-studies and they overall are defined as a feasibility study of an intervention. In table 1 there is reference to the sub-studies as being cross-sectional studies and also as a pre-post intervention. These study designs are contradictions unless you are undertaking some type of ecological study.

- there is reference on page 9 lines 238-9 to both qualitative and quantitative study input to the factors measured in the feasibility study. It leads one to believe that there should be a more widespread quantitative survey of CC diagnosed patients prior to the feasibility study.

- Given that the qualitative and quantitative studies are going to inform the feasibility study on the factors being measured, there does not appear to be much evidence for changing the already predetermined measures

- there is reference to a measure of morale as a secondary outcome but no reference to how that is being measured

- given that the intervention study is defined as a feasibility study then it would be expected that the objectives and outcomes should include some feasibility outcomes such as recruitment, retention, acceptability of measures and intervention, effect size etc.

- it is not clear what the mode of delivery of the surveys/questionnaire will be, online, person to person? There is reference to the survey data being captured on an EXCEL spreadsheet which appears to assume person to person but there is much more optimal ways to capture the data to minimize data entry error.

- power calculation appears to be generic and does not link to the expect effect size for the proposed study. However as a feasibility study the power calculation is not critical. Also retention or compliance is not accounted for in the calculation

- there is no reference to collection of any demographics or clinical characteristics of participants in the quantitative studies.

VERSION 1 - AUTHOR RESPONSE

FIRST REVIEW COMMENTS

1. **Comment:** Although a power calculation is provided for the quantitative phase, it would be helpful to clarify the anticipated effect size and provide justification for its choice.

Response: We appreciate the reviewer's valuable comment. We have clarified the assumptions underlying the power calculation and explicitly stated the anticipated effect size of this study (pages 12-13, lines 283-303). The sample size was calculated using a two-sample means formula, assuming a mean difference of 1 and a standard deviation of 2, which corresponds to a medium standardized effect size (Cohen's d = 0.5). The calculations were based on a two-sided significance level ($\alpha = 0.05$, $Z = 1.96$) and a power of 80% ($Z = 0.84$). This sample size will likely result in a medium effect size due to the nature of the intervention, previous literature, and the feasibility and practical impact:

i. **Nature of the Intervention**

- Health promoter-led initiatives are community-based, personalised, and culturally sensitive, which tend to yield moderate improvements in mental health and screening uptake.

ii. **Previous Literature**

- Studies on similar interventions (e.g., community health worker programs, psychoeducational support) often report moderate improvements in quality of life, anxiety, depression, and screening behaviour.

iii. **Feasibility and Practical Impact**

- A medium effect size is clinically meaningful and statistically detectable with a feasible sample size, especially in resource-limited settings like rural Eastern Cape.

2. **Comment:** As this is a feasibility study, the authors could also state explicitly that the primary aim is to assess feasibility parameters (e.g., recruitment, retention, adherence) rather than to achieve statistical power for hypothesis testing.

Response:

Thank you for the comment and advice. We have incorporated the advice in the manuscript (page 13, line 312-314): "The primary aim of this trial is to assess feasibility parameters (e.g., recruitment, retention, adherence) rather than to achieve statistical power for hypothesis testing".

3. **Comment:** More details are needed on the randomisation process—specifically, how cluster randomisation at the hospital level will be implemented, whether allocation concealment will be used, and how potential contamination between groups will be minimised.

Response: We appreciate the reviewer's insightful comments. Details on the randomisation and allocation concealment have been added to the manuscript (pages 13-15, lines 316-360). The added parts state that:

Since the intervention involves Health Promoter-Led Mental Wellness Initiatives, cluster randomising at the hospital level (rather than individual level) helps avoid contamination and aligns with real-world implementation. Step-by-Step Implementation is outlined below:

1. Define Clusters:

- Each of the two hospitals will serve as a cluster.
- For this study, clusters will be similar in terms of patient volume.

2. Randomisation Procedure:

- Researchers will use a computer-generated random sequence using STATA statistical software.
- Randomly assign clusters to either:
    - Intervention group: receives Health Promoter-Led Mental Wellness Initiatives.
    - Control group: receives standard care

3. Randomisation Unit:

- The unit of randomisation is the hospital, but outcomes are measured at the individual patient level.

To reduce selection bias:

- There will be centralised Randomisation:
- Researchers will use an independent statistician and data manager to generate and hold the randomisation list.
- Allocation is revealed only after baseline data collection is complete.
- While full blinding may not be feasible due to the nature of the intervention, outcome assessors and data analysts will be blinded to group allocation.

Minimising Contamination Between Groups

Contamination occurs when control group participants are exposed to the intervention. This will be achieved through multiple strategies:

i. Physical Separation

- The hospitals are geographically distant with no cross-over of staff.

ii. Staff Assignment

- Assign dedicated health promoters to intervention hospitals only.
- Avoid rotating staff between intervention and control sites.

iii. Communication Protocols

- Limit sharing of intervention materials or training across clusters until the study concludes.
- Use separate WhatsApp groups, training sessions, and supervision structures for each arm.

iv. Monitoring

- Conduct periodic checks to ensure fidelity to group assignments.
- Use surveys to track any unintended exposure to intervention content.

4. **Comment:** The protocol does not mention whether outcome assessors will be blinded to group allocation. This should be addressed to reduce potential bias in outcome measurement.

Response: Thank you for the comment and advice. The section on blinding of assessors has been added on page 14, line 340-341. The relevant statement states that: “While full

blinding may not be feasible due to the nature of the intervention, outcome assessors and data analysts will be blinded to group allocation".

5. **Comment:** While descriptive and inferential analyses are described, it would be valuable to specify how missing data will be handled and whether adjustments for multiple testing will be considered for secondary outcomes.

Response: Thank you for the comment. The section has been edited (page 20, lines 493-498) to state that: "All analyses will follow the intention-to-treat principle. We will examine patterns of missing data to understand their underlying mechanisms. If the data are missing at random, we will use multiple imputation techniques to reduce potential bias. Additionally, sensitivity analyses will be performed to assess the robustness of our findings against various assumptions regarding the missing data. For secondary outcomes, we will apply the Bonferroni correction to adjust for multiple testing, ensuring that we maintain control over the overall Type I error rate".

6. **Comment:** The description of the training for health promoters is clear; however, the protocol should also specify how the fidelity of motivational interviewing delivery will be monitored (e.g., supervision, checklists, session recordings).

Response: Thank you for the comment. The section has been edited to incorporate strategies for monitoring the fidelity of motivational interviewing delivery (page 19, lines 445-449). "The principal investigator will oversee the delivery of motivational interviewing, supervising the Health Promoters. All sessions will be recorded, and a checklist will be used to ensure that all topics are covered and that educational materials are provided".

7. **Comment:** The intervention lasts four months, with no planned long-term follow-up. This should be explicitly stated as a limitation, given that the sustainability of mental health improvements cannot be assessed.

Response: We appreciate your recommendation and would like to inform you that it has been acknowledged in the sections discussing the strengths and limitations of the study (page 3, line 61-63): "Since the study will only be conducted for four months and the intervention will be short-term, it will be difficult to establish sustainability of the improvements in mental health and quality of life among participants".

8. **Comment:** The protocol outlines clear procedures for obtaining informed consent and protecting data. However, given that participants with significant mental health issues (e.g., suicidal ideation) will be excluded, the authors could discuss the ethical and practical implications of excluding a potentially vulnerable subgroup.

Response: Thank you for the comment and advice. Page 15, lines 362-382 now clarifies this dilemma: Referral pathways to psychiatric or crisis services are in place for those mental health patients who are excluded, ensuring they are not left without support. Notwithstanding, patients living with cervical cancer who have suicidal ideation may benefit from integrated mental health services, and their exclusion could delay access to care. Researchers, therefore, further

acknowledge that there are ethical and practical implications of the exclusion criteria on patients with a mental health disorder:

i. Protection vs. Exclusion

- Ethical Justification: Excluding individuals with severe mental health conditions is primarily intended to protect them from potential harm, especially if the intervention is not equipped to manage acute psychiatric crises.

- Practical Limitation: However, this exclusion may inadvertently marginalise a subgroup that is both highly vulnerable and potentially in need of the very support the intervention aims to provide.

ii. Impact on Generalisability

- By excluding participants with significant mental health issues, the study may limit the applicability of its findings to the broader population of cervical cancer survivors, many of whom experience psychological distress.

- This could result in underestimating the true burden of mental health challenges in this population and overestimating the effectiveness of the intervention in real-world settings.

9. **Comment:** Since this is a feasibility study, it would be useful to pre-specify key feasibility outcomes (e.g., recruitment rate, session attendance, retention) and success criteria for proceeding to a full-scale trial.

Response: Thank you for your comment and advice. Page 16 -17, lines 385-389, has now added a statement and a Table: To assess whether the intervention is practical, acceptable, and scalable, six outcomes will be measured (Table 2):

Table 1: Pre-specified outcomes to measure practicality, acceptability and scalability of the intervention

Domain	Definition	Success criterion	Rationale
Recruitment Rate	Proportion of eligible cervical cancer patients who consent to participate.	≥70% of eligible participants recruited within the planned timeframe.	Indicates community interest and acceptability of health promoter-led initiatives.
Session Attendance	Proportion of participants attending ≥75% of scheduled	≥80% of participants meet attendance threshold.	Reflects engagement and feasibility of delivering the

	mental wellness sessions.		intervention in rural settings.
Retention Rate	Proportion of participants who complete all study assessments (baseline, post-intervention, follow-up).	≥85% retention across study phases	Ensures data completeness and indicates acceptability of study procedures
Health Promoter Fidelity	Adherence to intervention protocols by health promoters	≥90% of sessions delivered as per protocol (based on checklist or supervision logs)	Confirms intervention integrity and training effectiveness
Data Completeness	Proportion of participants with complete data for primary outcomes (e.g., mental health scores, quality of life)	≥90% completeness	Ensures reliability of outcome measures and feasibility of data collection tools
Acceptability and Satisfaction	Participant-reported satisfaction with the intervention (via post-session surveys or interviews)	≥80% report moderate to high satisfaction.	Gauges cultural and contextual fit of the intervention

SECOND REVIEWER COMMENTS

1. **Comment:** There is a lack of clarity as to how the quantitative study participants are going to be recruited.

Response: We appreciate the reviewer's insightful comments. Details on recruitment processes, randomisation, and allocation concealment are on (page 9, line 238-245). The statement is as follows: "Patients diagnosed with cervical cancer at both Nelson Mandela Academic Hospital and St. Elizabeth Hospital will be asked to consent to participate in a survey regarding their quality of life and mental health status, specifically focusing on screening for depression and anxiety. A total of 128 participants will be randomly selected and assigned to one of the two hospitals. These hospitals will also be randomly designated as either the control or intervention group. Each study arm will include an equal number of patients, with 64 participants allocated to each group".

2. **Comment:** Additionally, there is a reference to outpatients in the abstract and data management section, but not referred to elsewhere.

Response: Thank you for your observation. The term "outpatient" has been removed from both the abstract and data management sections (Page 2, line 42, and page 20, line 469) in the manuscript.

3. **Comment:** Background- page 4 lines 73-74, a statement is made about the increasing incidence and mortality in Africa, however it is not referenced. Later in the page, on lines 80-82 the statement is re-stated and this time has a reference. I recommend that the first statement is removed.

Response: Thank you for this observation. The statement (page 4, lines 74-75) has been removed as recommended.

4. Comment: Page 5 line 97 there is a statement that women living with HIV have an earlier onset of CC, I am unable to access the reference, but given risk factors for CC and HIV have some overlap it is not clear whether women living with HIV had the onset of CC while living with HIV or potentially prior to living with HIV?

Response: Reference has been added (page 4-5, line 96-97), and the statement has been rephrased (Women who have HIV have a higher chance of HPV infection and have a chance of developing early-onset cervical cancer while living with HIV.

5. **Comment:** Objectives -given that all participants (quantitative and qualitative) that are not clinical staff are women who have been diagnosed with CC, I am wary of including mental health status associated with CC screening in objective 1, unless the objective is slightly re-framed.

Response: Thank you for your observation. The objective has been modified to read: "To explore the perceived burden of mental health illnesses associated with cervical cancer, diagnosis, or treatment, targeting patients with cervical cancer and healthcare workers in the OR Tambo district" (page 8, line 198-200).

6. **Comment:** Methods- The qualitative Phase I appears to be well designed, However Phase II lacks some clarity, there is reference to 2 sub-studies and they overall are defined as a feasibility study of an intervention. In table 1 there is reference to the sub-studies as being cross-sectional studies and also as a pre-post intervention. These study designs are contradictions unless you are undertaking some type of ecological study.

Response: Thank you for the comment. The confusion has been cleared by eliminating the use of the term "cross-sectional study" and clarifying that it is a "longitudinal study", as it will include baseline, intervention, and subsequent evaluations at one-month intervals over a four-month period (Page 9, line 233 and page 11, line 263).

7. **Comment:** There is reference on page 9 lines 238-9 to both qualitative and quantitative study input to the factors measured in the feasibility study. It leads one to believe that there

should be a more widespread quantitative survey of CC diagnosed patients prior to the feasibility study.

Response: We appreciate the reviewer's observation. The statement was not intended to suggest that a large-scale quantitative survey of patients diagnosed with cervical cancer was conducted prior to the intervention study. Rather, it refers to the incorporation of both qualitative insights (from interviews and discussions) and quantitative indicators (such as existing clinical and demographic data) to inform the identification of relevant feasibility factors. The wording has been revised to clarify this point and avoid any misunderstanding (page 10, lines 248-250).

8. **Comment:** Given that the qualitative and quantitative studies are going to inform the feasibility study on the factors being measured, there does not appear to be much evidence for changing the already predetermined measures.

Response: We appreciate the reviewer's observation. The intention of integrating qualitative and quantitative components prior to the intervention study is not to alter the predetermined measures, but rather to refine the contextual understanding of how these measures will be applied within the study population, to ensure their practical relevance and acceptability without altering their core structure.

9. **Comment:** There is a reference to a measure of morale as a secondary outcome, but no reference to how that is being measured

Response: We appreciate the reviewer's observation. The statement has been revised. Secondary outcomes will include attendance rate, retention, and data completeness at intervention sessions (Page 10, lines 246-247).

10. Comment: Given that the intervention study is defined as a feasibility study, then it would be expected that the objectives and outcomes should include some feasibility outcomes such as recruitment, retention, acceptability of measures and intervention, effect size, etc.

Response: Thank you for the comment and advice. Page 16-17, line 386-390, has now added a statement and a Table:

To assess whether the intervention is practical, acceptable, and scalable, six outcomes will be measured (Table 2):

Table 2: Pre-specified outcomes to measure practicality, acceptability and scalability of the intervention

Domain	Definition	Success criterion	Rationale
Recruitment Rate	Proportion of eligible cervical	≥70% of eligible participants recruited	Indicates community interest and

	cancer patients who consent to participate.	within the planned timeframe.	acceptability of health promoter-led initiatives.
Session Attendance	Proportion of participants attending ≥75% of scheduled mental wellness sessions.	≥80% of participants meet attendance threshold.	Reflects engagement and feasibility of delivering the intervention in rural settings.
Retention Rate	Proportion of participants who complete all study assessments (baseline, post-intervention, follow-up).	≥85% retention across study phases	Ensures data completeness and indicates acceptability of study procedures
Health Promoter Fidelity	Adherence to intervention protocols by health promoters	≥90% of sessions delivered as per protocol (based on checklist or supervision logs)	Confirms intervention integrity and training effectiveness
Data Completeness	Proportion of participants with complete data for primary outcomes (e.g., mental health scores, quality of life)	≥90% completeness	Ensures reliability of outcome measures and feasibility of data collection tools
Acceptability and Satisfaction	Participant-reported satisfaction with the intervention (via post-session surveys or interviews)	≥80% report moderate to high satisfaction.	Gauges cultural and contextual fit of the intervention

11. **Comment:** It is not clear what the mode of delivery of the surveys/questionnaire will be, online, person to person?

Response: Thank you for the comment. All interviews and questionnaires will be undertaken/administered in person. This has now been clarified in the Methods section of the manuscript. This face-to-face approach enables direct engagement, ensuring that participants feel at ease and can ask questions for clarification as needed. By delivering the materials personally, the study aims to foster a supportive environment that encourages honest and thoughtful responses (Page 18, lines 433-434).

12. **Comment:** There is reference to the survey data being captured on an EXCEL spreadsheet which appears to assume person to person but there is much more optimal ways to capture the data to minimise data entry error.

Response: We appreciate the reviewer's comment regarding data capture. While the initial draft mentioned using Excel for data entry, survey data will be captured electronically using a secure data management platform with built-in validation checks to ensure accuracy and minimise data entry errors. The manuscript has been updated to reflect this improved approach. (page 20, line 476-478).

13. **Comment:** - power calculation appears to be generic and does not link to the expect effect size for the proposed study. However, as a feasibility study, the power calculation is not critical. Also retention or compliance is not accounted for in the calculation.

Response: We appreciate the reviewer's valuable comment. We have clarified the assumptions underlying the power calculation and explicitly stated the anticipated effect size of this study (pages 12-13, lines 284-304). The sample size was calculated using a two-sample means formula, assuming a mean difference of 1 and a standard deviation of 2, which corresponds to a medium standardized effect size (Cohen's d = 0.5). The calculations were based on a two-sided significance level ($\alpha = 0.05$, $Z = 1.96$) and a power of 80% ($Z = 0.84$). This sample size will likely result in a medium effect size due to the nature of the intervention, previous literature and the feasibility and practical impact:

i. **Nature of the Intervention**

- Health promoter-led initiatives are community-based, personalised, and culturally sensitive, which tend to yield moderate improvements in mental health and screening uptake.

ii. **Previous Literature**

- Studies on similar interventions (e.g., community health worker programs, psychoeducational support) often report moderate improvements in quality of life, anxiety, depression, and screening behaviour.

iii. **Feasibility and Practical Impact**

- A medium effect size is clinically meaningful and statistically detectable with a feasible sample size, especially in resource-limited settings like the rural Eastern Cape.

14. **Comment:** - There is no reference to collection of any demographics or clinical characteristics of participants in the quantitative studies.

Response: The WHO-QoL-BREF instrument, as outlined in Appendix 7, will include a comprehensive set of demographic and clinical characteristics. These details, described in items 1-6, will provide valuable insights into the participants' backgrounds and health conditions. (Page 18, line 429-430).
